# Simulations to benchmark time-varying connectivity methods for fMRI

**DOI:** 10.1371/journal.pcbi.1006196

**Published:** 2018-05-29

**Authors:** William Hedley Thompson, Craig Geoffrey Richter, Pontus Plavén-Sigray, Peter Fransson

**Affiliations:** 1 Department of Psychology, Stanford University, Palo Alto, California, United States of America; 2 Department of Clinical Neuroscience, Karolinska Institutet, Stockholm, Sweden; 3 BCBL, Basque Center on Cognition, Brain and Language, Donostia, Spain; Ghent University, BELGIUM

## Abstract

There is a current interest in quantifying time-varying connectivity (TVC) based on neuroimaging data such as fMRI. Many methods have been proposed, and are being applied, revealing new insight into the brain’s dynamics. However, given that the ground truth for TVC in the brain is unknown, many concerns remain regarding the accuracy of proposed estimates. Since there exist many TVC methods it is difficult to assess differences in time-varying connectivity between studies. In this paper, we present *tvc_benchmarker*, which is a Python package containing four simulations to test TVC methods. Here, we evaluate five different methods that together represent a wide spectrum of current approaches to estimating TVC (sliding window, tapered sliding window, multiplication of temporal derivatives, spatial distance and jackknife correlation). These simulations were designed to test each method’s ability to track changes in covariance over time, which is a key property in TVC analysis. We found that all tested methods correlated positively with each other, but there were large differences in the strength of the correlations between methods. To facilitate comparisons with future TVC methods, we propose that the described simulations can act as benchmark tests for evaluation of methods. Using *tvc_benchmarker* researchers can easily add, compare and submit their own TVC methods to evaluate its performance.

## Author summary

Time-varying connectivity attempts to quantify the fluctuating covariance relationship between two or more regions through time. In recent years, it has become popular to do this with fMRI neuroimaging data. There have been many methods proposed to quantify time-varying connectivity, but very few attempts to systematically compare them. In this paper, we present *tvc_benchmarker*, which is a python package that consists of four simulations. The parameters of the data are justified on fMRI signal properties. Five different methods are evaluated in this paper, but other researchers can use tvc_benchmarker to evaluate their methodologies and their results can be submitted to be included in future reports. Methods are evaluated on their ability to track a fluctuating covariance parameter between time series. Of the evaluated methods, the jackknife correlation method performed the best at tracking a fluctuating covariance parameter in these four simulations.

## Introduction

Time-varying connectivity (TVC) is being applied to an increasing number of topics studying the brain’s networks. Topics that have been explored with TVC include development [1], various pathologies [2, 3], affect [4], attention [5], levels of consciousness [6], and temporal properties of the brain’s networks [7–9]. There are many concerns raised regarding methodological issues. These issues span biased variance [10, 11], movement artefacts [12], and appropriate statistics [13, 14].

Methods used to derive TVC estimates are as diverse as its range of applications. Examples of different methods include: the sliding window method, sometimes tapered [15], multiplication of temporal derivatives [16], methods using Euclidean distance between spatial configurations [8], k-means clustering methods [7, 17], eigenconnectivities [18], point process methods [19, 20], Kalman filters [21, 22], flexible least squares [23], temporal ICA [24], sliding window ICA [25], dynamic conditional correlation [26], phase differences [27] wavelet coherence [4], hidden Markov models [28], and variational Bayes hidden Markov models [29]. This list of TVC methods is not exhaustive, and even more methods can be found in the literature.

While these methods and their applications may offer new insights into the functions of the brain and cognition, it becomes difficult to compare results when different studies use different methods to estimate brain dynamics. Each method is often introduced and evaluated by the authors’ own simulations, empirical demonstrations, and/or theoretical arguments. However, apparent differences in time-varying connectivity in different studies may have been influenced, or even caused, by differences in the underlying methodology used to derive connectivity estimates.

In order to maximize reproducibility of reported findings, it is important that comparisons of proposed TVC methods can be made with a common set of simulations. To this end, we have developed four simulations that aim to show how well results from different TVC methods correlate with each other and evaluate their performance of tracking time varying covariance. The proposed methods and simulations are included in the Python package *tvc_benchmarker*, (available at www.github.com/wiheto/tvc_benchmarker). Researchers can evaluate their own TVC methods in *tvc_benchmarker*. The software also allows for new methods to be submitted to us for inclusion in future reports. Here we demonstrate the functionality and results obtained by *tvc_benchmarker* by evaluating the performance of the following five methods: sliding window (SW), tapered sliding window (TSW), spatial distance (SD), jackknife correlation (JC), and multiplication of temporal derivatives (MTD).

## Methods

### Software used

All methods for TVC derivation were implemented in Teneto v0.2.7b [8]. Bayesian statistics for evaluating performance of TVC methods were calculated in PyMC3 V3.1 [30], simulations and analysis were done using Numpy V1.13.1 [31], Scipy V0.19.1 [32], and Pandas V0.19.2. Matplotlib V2.0.2 [33] and Seaborn V0.7.1 [34] were used for figure creation.

### Time-varying connectivity methods

As discussed in the introduction, the list of published TVC methods that are designed to be applied to fMRI imaging data is long. In an ideal world all methods will be contrasted under the same conditions such that an evaluation of those methods that give appropriate results can be performed. However, it was not our intention to provide a complete comparison of all published methods. Instead we have made all simulation tools freely available so that researchers can evaluate their own TVC methods. Before describing the simulations and the results, we provide a brief overview of the five methods that are evaluated in this article.

#### Sliding window (SW)

The SW method is one of the most commonly used methods to estimate TVC. The sliding window method uses a continuous subsection of the data, estimates the degree of correlation (Pearson correlation), slides the window one step in the time series, and repeats. This creates a smooth connectivity time series as neighbouring estimates of connectivity share all but two data points. The SW method is based on the assumption that nearby temporal points are helpful to estimate the covariance. In our simulations, two different window lengths were chosen: 15 and 29 (when necessary these are referred to as SW-15 and SW-29). Given the common choice of a time resolution (TR) of 2 seconds in fMRI, this results in a window length of 30 and 58 seconds which touches the upper and lower bound for rule-of-thumb window lengths that has been suggested [35]. The reason for choosing odd number window lengths is to ensure that the center of each window corresponds to a specific time-point.

#### Tapered sliding window (TSW)

The TSW method can be described as a weighted Pearson correlation where the weights are set to zero except for the data points residing inside the window. This procedure is identical to the SW method except that a larger weight is placed on time points closer to the centre of the window (*t*). Often, the weights are distributed according to a Gaussian distribution centred at *t*. In our simulations using the TSW method, we used a Gaussian distribution with a variance of 10 time-points. The window lengths were the same as for the SW method (centered at *t*) and referred to as TSW-15 and TSW-29. See also [15] for an example of usage of the TSW method.

#### Spatial distance (SD)

In the sliding window methods, temporally adjacent data points are used to estimate the covariance. An alternative is to use time points that have similar spatial profiles. There are two steps to this method: first, a weight vector is calculated for each time point using the spatial distance between all other time points; second, a weighted Pearson correlation is used to derive the connectivity estimate at *t*.

To calculate the weight vector for *t* (*w*_*t*_), each weight is based on the distance of the spatial dimensions and another time point. In functional neuroimaging data the “spatial dimensions” correspond to the amplitude of the signal for the voxels or regions of interest. While the weights can be derived in multiple ways, [8] took the inverse of a distance function between the spatial dimension amplitudes at *t* and for each other time point (*u*):

$$\begin{array}{c} w_{u}^{\left(t\right)}=\frac{1}{D_{t,u}}\;t\neq u \end{array}$$ (1)

where the Euclidean distance was selected for *D*. This entails that time points that have a similar activation profiles close to *t* will get larger weights. The weight vectors are each subsequently scaled between 0 and 1. The “self weights” ($w_{t}^{\left(t\right)}$) are set to 1. Each time point gets its own weight vector (*w*_*t*_) which is the length of the time series.

After the weight vector has been calculated, the connectivity estimate at *t* is the weighted Pearson correlation where each time point is weighted by *w*^(*t*)^. This entails that points that are spatially close are considered. For more details of the SD method, see [8]. See [36] for a detailed discussion how the SD method differs in its assumptions from the sliding window methods.

There is an important difference in [8] and the simulations here. [8] uses all regions of interest (not just two time series) to calculate the weight vector. As there are only two time series in all the simulations in this paper, this might be considered closer to a bivariate version of the SD method where each edge has its own collection of weight vectors (i.e. the bivariate SD method will have a weight vector for each edge and time point while the multivariate SD method will have a weight vector for each time point). S1 Appendix demonstrates that, on fMRI data, there is a large correlation between the bivariate and multivariate methods (mean Spearman rank (*ρ*): 0.76 (from 38,503 edges)).

#### Jackknife correlation (JC)

The JC method has previously been shown on electrocorticographic data for single trial coherence and Granger causality [37]. To the best of our knowledge, the jackknife correlation method has not yet been utilized in the TVC literature. Thus, we provide a more detailed description of its logic and workings. The jackknife correlation method is outlined in more detail and contrasted to a binning approach (which is akin to the sliding window method) in [37]. The JC method, when applied to single time point covariance estimates of signals *x* and *y* at *t* computes the Pearson correlation between the two signals using all time points in *x* and *y* with the exception of *x*_*t*_ and *y*_*t*_:

$$\begin{array}{c} JC_{t}=-(\frac{\sum_{i}^{T}\left(x_{i}-{\bar{x}}_{t}\right)\left(y_{i}-{\bar{y}}_{t}\right)}{\sum_{i}^{T}{\left(x_{i}-{\bar{x}}_{t}\right)}^{2}\sum_{i}^{T}{\left(y_{i}-{\bar{y}}_{t}\right)}^{2}})\;i\neq t \end{array}$$ (2)

Of note, the inclusion of the minus sign in the equation above is to correct for the inversion caused by the leave-n-out process (see below). The ${\bar{x}}_{t}$ and ${\bar{y}}_{t}$ are the expected values, excluding data at time point *t*:

$$\begin{array}{c} {\bar{x}}_{t}=\frac{1}{T-1}\sum_{i}^{T}x_{i}\;i\neq t \end{array}$$ (3)

**Fig 1:**
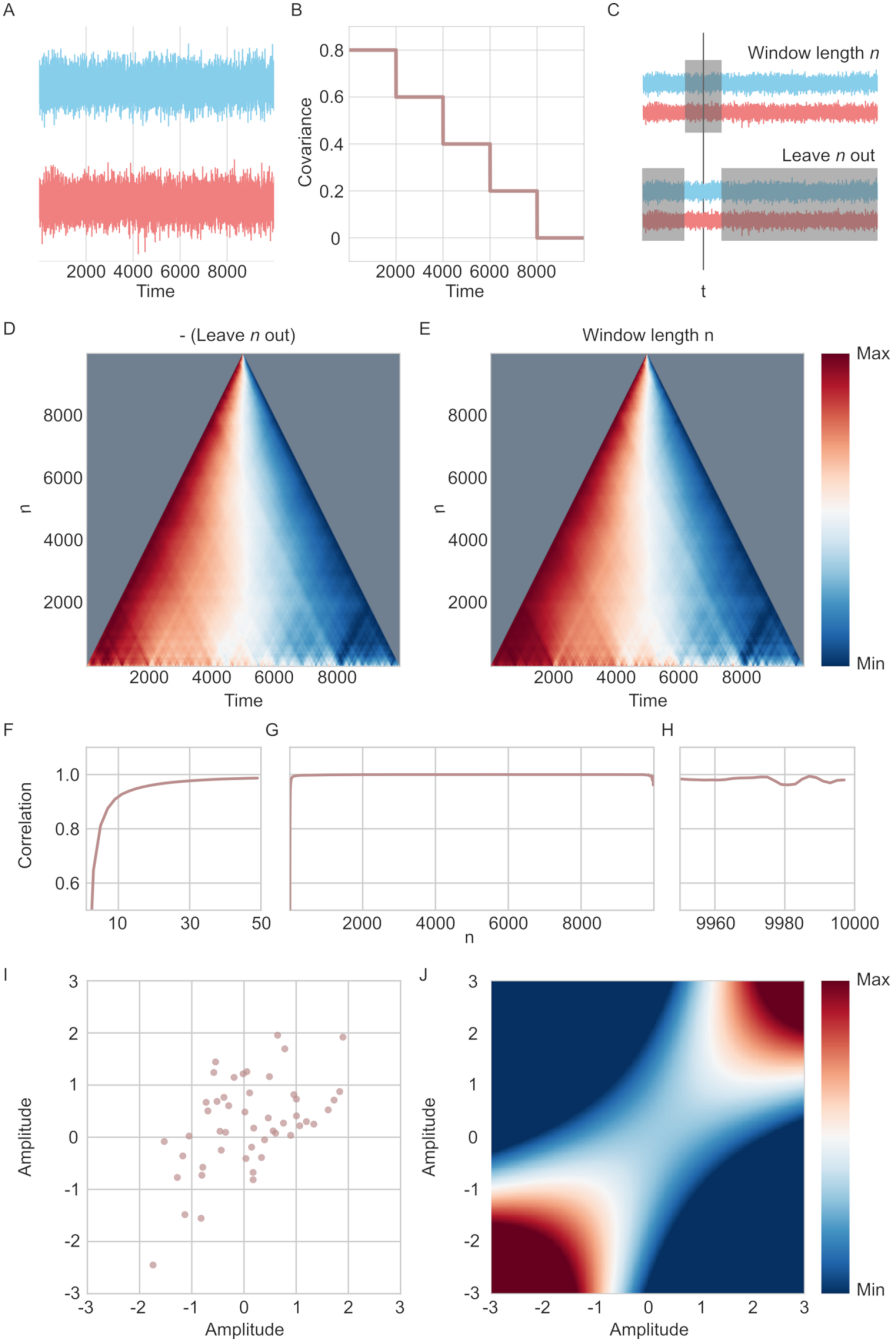
Illustration of jackknife correlation and leave-*n*-out/window-length-*n* symmetry. (A) Two time series drawn from a multi-variate Gaussian distribution, stretching over 10,000 time points with their covariance parameter changing every 2,000th time point. (B) The covariance parameter of the two time series in A. (C) Depiction of how the window-length-n and leave-*n*-out relate to each other. Shaded region indicates time points used in the correlation estimate at time point *t*. (D) The correlation estimate per time point for varying n of the leave-*n*-out method (correcting for the inversion by multiplying with -1). The time series for each *n* is scaled between 0 and 1. (E) Same as D, but for the window-length-*n* method. (F-H) Correlation between the time series of connectivity estimates for window-length-*n* and leave-*n*-out methods for different values of *n*. (F) Shows *n* between 1-50 (G) Shows *n* over the entire time series. (H) Shows *n* between 9,950 and 10,000. (I) The correlate of the amplitude of the two time series. 49 time points were sampled from a multivariate Gaussian distribution with a covariance of 0.5. (J) Illustration of the jackknife correlation estimate for different possible values, relative to the 49 time points in I.

To demonstrate the JC method, 10,000 time points were drawn from a multivariate Gaussian distribution with a mean of 0 and a variance of 1 to generate the two time series shown in Fig 1A. Additionally, the time series were constructed so that the covariance between the two varied as a function of time. For the first 2,000 time points, the covariance was set to 0.8 and then further decreased in steps of 0.2 for every 2,000th time point (Fig 1B).

The *relative* connectivity time series are similar (but inverted) for a leave-*n*-out compared to a window-length-*n* methods (Fig 1C). The JC method corresponds to the case when *n* = 1, i.e. a leave-1-out approach after correcting for the inversion (all leave-*n*-out estimates are multiplied by -1 to correct for the inversion in Fig 1). All possible choices of *n* were computed for the leave-n-out method: (i.e. a leave-1-out to a leave-9,998-out. The window-of-length-n was computer for n = 2 to n = 9,998 (see Fig 1D and 1E). Note that the window-length method could be 9,999 and 10,000 but was not used as the leave-n-out method cannot do this.

As shown in Fig 1G, the Spearman correlation between the two methods is close to 1 for various choices of *n*. However, their correspondence in covariance estimates between the two methods departs at the tails (Fig 1F and 1H). These deviations occur for two reasons: (1) when *n* is very small it implies there is little data to work with for the window-length-*n* method. On the other hand, low values of *n* do not hamper the performance of the leave-*n*-out method. (2) Large values of *n* will result in few estimates for covariance for each time point, which makes the correlation between the two methods less stable. In sum, while it is impossible to create estimates for window-of-length-1, it is however possible to use a leave-1-out method as an approximation for a window-length-1 due to the symmetry between the two methods.

When estimating the TVC, the two major aims are to accurately measure the covariance and to be sensitive to changes in the covariance. In the case of the leave-1-out (i.e. the jackknife correlation) approach, we achieve a unique connectivity estimate per time point that is more reliable than using a smaller window size (due to the fact that more data is used). Usually, the SW method has to find a balance between the two aims. In this respect, the JC method is an optimal sliding window method as it does not have to compromise between temporal sensitivity on the one hand and accuracy on the other.

The time point based TVC estimate obtained with the JC method should be interpreted as the relative difference in connectivity at any particular data point compared to all other data points in the time series. This is because the covariance for each data point is estimated based on its relationship to all other data points. To illustrate this effect, consider the 49 data points randomly sampled from a Gaussian distribution with a mean of 0 and a covariance of 0.5 as shown in Fig 1I. If we assume that the 49 time points are used to compute the JC estimate for the covariance for a 50th time point, the value of this new data point will have no impact on its JC covariance estimate because the other 49 points are used. What the 50th time point does is change the JC estimate for the other 49 points. This means that the relative position of the 50th point changes in relation to the rest of the time series. The standardized JC estimate of covariance for all possible values of the 50th time point is shown in Fig 1J. What this example shows is that an individual JC estimate has little meaning by itself and only becomes meaningful relative to the other JC estimates in the time series.

When using the JC method to estimate TVC, it is important to keep in mind that it leads to a compression of the variance. Furthermore, the amount of compression is proportional to the length of the time series. It is often helpful to scale or standardize the connectivity time series derived by the JC method before any subsequent analysis. Finally, while a Pearson correlation was used in this study for the JC, it is possible to use other correlation methods such as the Spearman Rank instead.

#### Multiplication of temporal derivatives (MTD)

The MTD approach to estimate TVC was first introduced in [16]. In brief, the multiplication of temporal derivatives method first computes the temporal derivative of a time series as:

$$\begin{array}{c} df_{i}=x_{i,t}-x_{i,t-1} \end{array}$$ (4)

Next, the coupling between the signal sources *i* and *j* is defined as the product of the two derivatives *df*_*i*_ and *df*_*j*_ for each time point *t*, divided by the product of the standard deviation for *df*_*i*_ and *df*_*j*_:

$$\begin{array}{c} MTD_{i,j,t}=\frac{df_{i,t}df_{j,t}}{\sigma_{df_{i}}\sigma_{df_{j}}} \end{array}$$ (5)

The MTD method is often used together with a smoothing function in the form of a window function. In our simulations, a window length of 7 was chosen, since this was considered optimal in [16].

#### Post-processing for TVC estimates

After each of the TVC methods were applied to the simulated data a Fisher transform was applied to the connectivity time series (except for the MTD method). To illustrate the variance compression that results from the JC method, the TVC for the JC method was not standardized in Simulation 1.

The SW, TSW and MTD methods should all be greatly affected by any autocorrelation existing in the signal as they all use windows of neighboring time points. The SD and JC methods are more robust to affects of autocorrelation since these two methods are permutation-invariant (i.e. they will return the same estimates even if the order of the time series is shuffled), unlike SW, TSW, and MTD.

### Simulations

This section provides an overview of the simulations that are conducted and the general methodology used. See each simulation’s subsection in the results section for full details of each simulation.

To compare accuracy and performance for the five TVC methods, we performed four different simulations. The first simulation investigated the similarity of the different TVC methods by correlating their respective connectivity estimates. The second simulation targeted how well the different methods were able to track a fluctuating covariance parameter. The third simulation tested how robust the estimated fluctuating covariance is when the mean of the time series fluctuates, mimicking the haemodynamic response function. The forth simulation considered whether TVC methods can accurately track abrupt changes in covariance.

All simulations considered two time series each consisting of 10,000 samples generated from multivariate Gaussian distributions. At each time point, the covariance between the time series could vary (see below). A full account of all model assumptions made as well as a justification for our model parameter settings for the four simulations models used in the present study are given in S2 Appendix.

Simulations 2, 3, and 4 all consisted of a fluctuating covariance parameter (*r*_*t*_) that was used to generate the covariance between the time series. TVC methods were evaluated based on their ability to track the *r*_*t*_ parameter. How *r*_*t*_ was generated could vary for different simulations. In simulation 2, *r*_*t*_ varied throughout the time course based on a normal distribution. The simulation was run multiple times allowing for different autocorrelation of *r*_*t*_ through time. In simulation 3, *r*_*t*_ varied in the same way as simulation 2 but it was applied to time series that had a non-stationary mean that mimicked a HRF. This simulation was also run multiple times with different autocorrelations. In simulation 4, *r*_*t*_ varied based on two different “states” that lasted for varying amounts of time. This method was run two times when states could be short (2-6 time points long) or long (20-60 time points long). By evaluating the correlation of different TVC methods with each simulation’s *r*_*t*_, we can evaluate which time varying properties a method is sensitive to.

Simulation 1-3 have all their parameters justified on empirical data in S2 Appendix. Simulation 4 has its state lengths based on what has been identified by different TVC studies. It is important to stress that these different state lengths may have been identified due to the methods which were used and may not reflect real dynamic properties.

### Statistics

In principle, it is possible to simply correlate the results from the different TVC methods with the *r*_*t*_ values of each simulation to statistically evaluate their performance. However given the inherent, but known, uncertainty in *r*_*t*_, we deemed it was appropriate to create a statistical model which accounts for this uncertainty. Thus, for each TVC method, a Bayesian statistical model was created to evaluate the relationship between the TVC estimate and the signal covariance.

The Bayesian model aims to predict *y*, which is the vector of the known sampled covariances (i.e. *r*_*t*_) with *x*, which is the connectivity estimate for each TVC model.

$$\begin{array}{c} y_{i}∼N\left(\mu_{i},\sigma \right)\\ \mu_{i}=\alpha +\beta x_{i}\\ \alpha ∼N(0,1)\\ \beta ∼N(0,1)\\ \sigma ∼N_{\text{half}}\left(0,1\right) \end{array}$$ (6)

All TVC estimates and the values of *r*_*t*_ were standardized prior to calculating the models with a mean of zero and standard deviation of one. This was done to facilitate the interpretation of the posterior distribution parameter *β*. The different TVC methods vary in the number of time points estimated (e.g. the beginning and end of the time series cannot be estimated with the sliding window method). In order to facilitate model comparison between methods, we restrained the simulations to include only the time points that had estimates from all TVC methods (i.e the limit was set by the SW and TSW methods which can estimate the covariance for 9,972 out of 10,000 time points).

The statistical models were estimated through 5,500 draws from a Markov Chain Monte Carlo (MCMC) with a No-U-Turn Sampler [38] sampler implemented in pymc3. The first 500 samples were burned.

The statistical models for the different TVC methods can be contrasted in two ways: (1) model comparison by examining the model fit; (2) by comparing the posterior distribution of *β* for the different TVC methods. To evaluate the model fit, the Watanabe-Akaike information criterion (WAIC, [39]) was used. The posterior distribution of *β* illustrates the size and uncertainty of the relationship between *x* and *y*. To aid the interpretation of these results for readers unfamiliar with Bayesian statistics, the mode of the distribution corresponds approximately to a maximum-likelihood estimated *β* value in a linear regression (if uniform priors are used for the parameters the posterior mode and the maximum-likelihood estimator would have been exactly the same).

In simulation 1, the different TVC estimates are compared with each other to evaluate how similar these estimates are. To do this, a Spearman correlation is used to evaluate the relationship.

## Results

### Simulation 1

The first simulation aimed to quantify the similarity of the different TVC time series estimates. If two TVC methods are strongly correlated, this is a positive sign that they are estimating similar aspects of the evolving relationship between time series. A negative correlation between two methods would suggest that they do not capture the same dynamics of the signal.

In this simulation we created two time series (*X*), each consisting of 10,000 time points in length. The time series were constructed by:

$$\begin{array}{c} X_{t}=\alpha X_{t-1}+\epsilon \end{array}$$ (7)

The autocorrelation with lag of 1 is determined by *αX*_*t*−1_ and the covariance at *t* is determined by *ϵ*. *ϵ* was sampled from a multivariate Gaussian distribution (N):

$$\begin{array}{c} \epsilon ∼N\left(\mu ,\Sigma \right) \end{array}$$ (8)

where *μ* is the mean and Σ being the covariance matrix of the multivariate Gaussian distribution. Both time series were set to have a mean of 0, variance of 1 and a covariance of 0.5. In summary:

$$\begin{array}{c} \mu =0,0\\ \Sigma =(\begin{array}{cc} 1 & 0.5\\ 0.5 & 1 \end{array}) \end{array}$$ (9)

**Fig 2:**
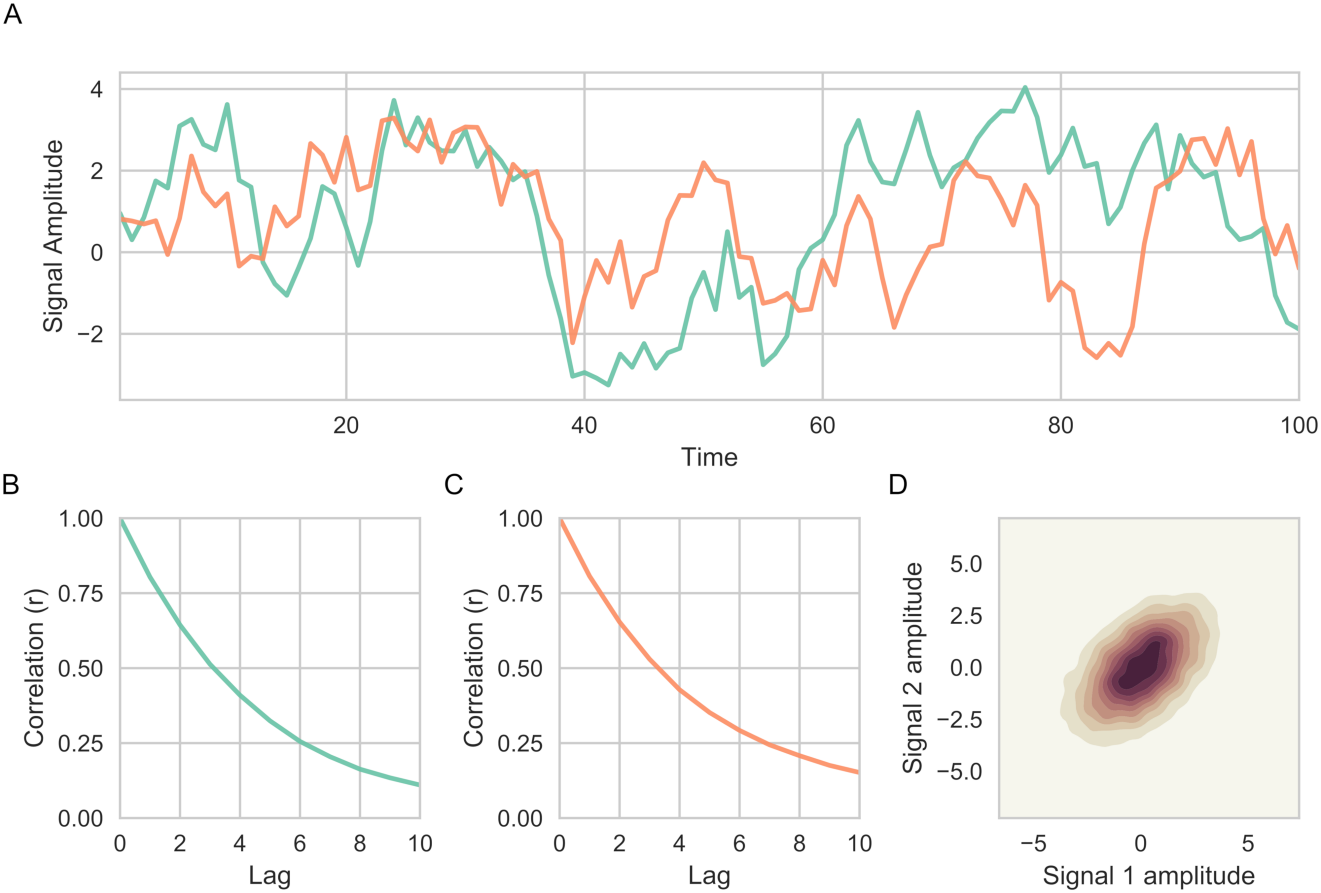
Simulated data in Simulation 1. (A) Two correlated time series were generated (a total of 10,000 time points were simulated, only the first 100 time points shown in the figure for illustration purposes). (B-C) Autocorrelation of both time series (colors corresponding to respective time series given in (A)) for up to 10 lags. (D) Kernel density estimation illustrating the covariance between two time series (*r* = 0.51).

The autoregressive parameter *α* controls the size of the autocorrelation in relation to the preceding time point (i.e. the proportion of the previous time point that is kept). Here, it was set to 0.8 which was deemed to be an appropriate degree of autocorrelation for BOLD time series (see S2 Appendix). A portion of the two simulated time series is found in Fig 2A together with the plots of their respective autocorrelation (Fig 2B and 2C) and a plot of the correlation between the two time series (Fig 2D).

**Fig 3:**
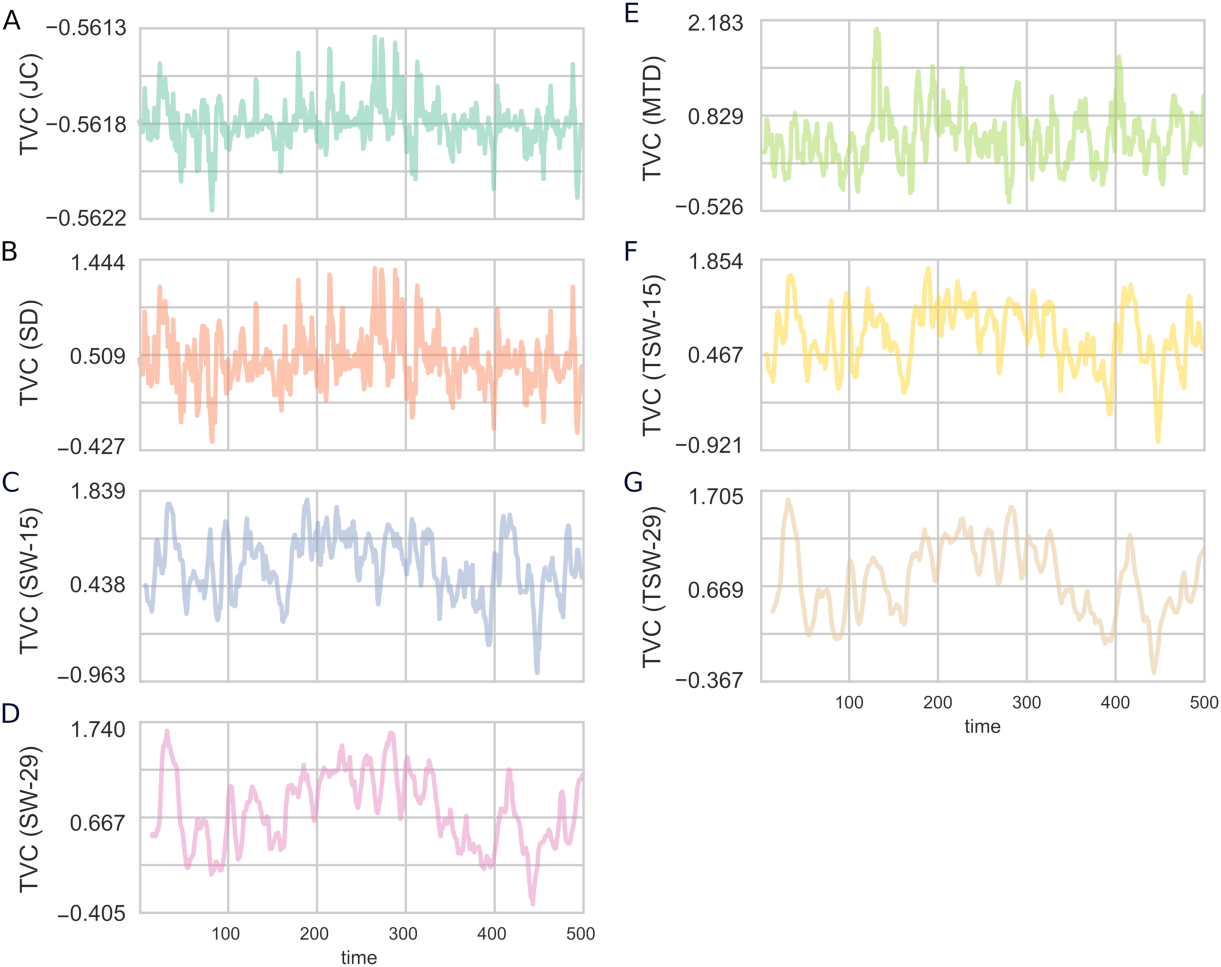
Time-varying connectivity estimates for Simulation 1. The TVC methods: (A) JC; (B) SD; (C) SW-15; (D) SW-29; (E) MTD; (F) TSW-15; (G) TSW-29. Only the first 500 time points are shown for illustration purposes.

The resulting connectivity time series for the different TVC methods when applied to the simulated data is shown in Fig 3. From Fig 3, several qualitative observations can be made about the methods. Firstly, there was a very strong similarity between the SD and JC methods, despite the fact that they consist of quite different assumptions. Further, the SD, JC, and MTD methods were all able to capture considerably quicker transitions than the SW and TSW methods. The long window lengths (SW-29 and TSW-29) were smoother than the SW-15 and TSW-15 methods. Finally, the variance of the JC method was considerably smaller than all other methods, illustrating the variance compression as previously discussed.

**Fig 4:**
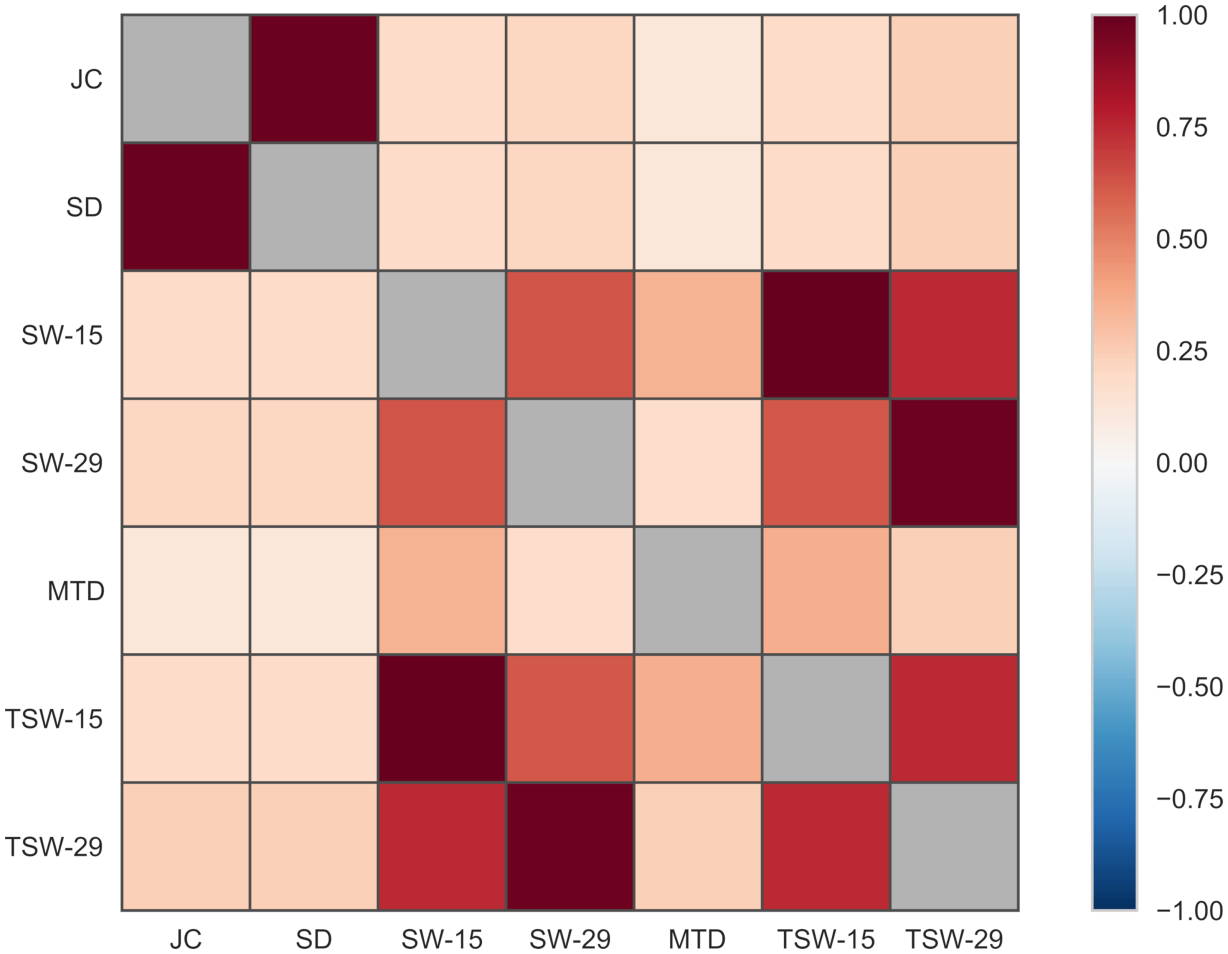
The degree of similarity of functional connectivity estimates for all tested TVC methods computed with the Spearman correlation coefficient in Simulation 1.

To assess the degree of similarity of the estimates of functional connectivity time series obtained from all TVC methods, a Spearman correlation was computed for each TVC method pairing (Fig 4). The connectivity time series estimates from all methods correlated positively with each other (Fig 4). Some methods showed strikingly strong correlations (SD & JC: 0.976; SW-15 & TSW-15: 0.999; SW-29 & TSW-29: 0.978). Between the different window lengths the correlation was slightly smaller (SW: 0.644; TSW: 0.755). The lowest correlation was found between the JC and MTD methods (*ρ* = 0.138).

The results from Simulation 1 showed that the connectivity estimates provided by the tested methods are, to a varying extent, correlated positively with each other. It also illustrated how the different methods differ in their resulting smoothness of the connectivity time series. The results from this simulation cannot validate whether any TVC method is superior to any other, it merely highlights which methods produce similar connectivity time series.

### Simulation 2

In Simulation 1, it was not possible to evaluate how well the different TVC methods perform. To evaluate the performance, the simulated data must change its covariance over time and how this changes must be known beforehand. The aim of this simulation was to see how well the derived TVC estimates can infer the covariance that the data was sampled from when the covariance is fluctuating.

Two time series were generated (*X*). Each time point *t* is sampled from a multivariate Gaussian distribution:

$$\begin{array}{c} X_{t}∼N\left(\mu ,\Sigma_{t}\right) \end{array}$$ (10)

where the covariance matrix was defined as:

$$\begin{array}{c} \Sigma_{t}=(\begin{array}{cc} \sigma & r_{t}\\ r_{t} & \sigma \end{array}) \end{array}$$ (11)

and where the variance, *σ* = 1, was set to 1. At each time point, *r*_*t*_ was sampled from another Gaussian distribution:

$$\begin{array}{c} r_{t}∼N\left(\mu_{r},\sigma_{r}\right) \end{array}$$ (12)

The mean of the time series (*μ*) was set to 0, the mean of the covariance (*μ*_*r*_) was set to 0.2. The simulation was run three times where the parameter for the variance of the fluctuating covariance (*σ*_*r*_) was set to three different values {0.08, 0.1, 0.12}. This ensured that the different TVC methods are robust to different variances of connectivity changes.

The covariance at time (*r*_*t*_) was sampled from a Gaussian distribution. Each time point received a new value of *r*_*t*_. This allowed us to compare each TVC method’s connectivity estimate in relation to the time varying covariance parameter *r*_*t*_. Note, that at each time point the relationship between the two time series is dictated by a single realization from a Gaussian distribution where *r*_*t*_ is the covariance. Thus, we should not expect the connectivity estimate from any method to correlate perfectly with *r*_*t*_. However, it is possible to compare which method correlate better or worse with *r*_*t*_ to evaluate the overall performance.

The above model will have a temporally fluctuating covariance. It fails to include any autocorrelation in the time series. Not accounting for this may bias the results for some of the tested methods that utilize nearby temporal points to assist estimating the covariance. Merely adding an autocorrelation, like in Simulation 1, will also increase the covariance between the two time series and this will not be tracked by *r*_*t*_. To account for this, we placed a 1-lag autoregressive model for the fluctuating covariance at *r*_*t*_:

$$\begin{array}{c} r_{t}=\alpha r_{t-1}+\epsilon \end{array}$$ (13)

$$\begin{array}{c} \epsilon ∼N\left(\mu_{r},\sigma_{r}\right) \end{array}$$ (14)

Where *α* is the autocorrelation parameter. The values for *μ*_*r*_ and *σ*_*r*_ were the same as above. When *t* = 1, *ϵ* was set to 0.

This revised formulation of our simulation model allowed for the covariance to fluctuate, but with an added autocorrelation on the covariance parameter. In simulation 2, three different settings of the parameter *α* were used (*α* = 0, 0.25, 0.5). When *α* = 0 it is equivalent to the original model outlined above with no autocorrelation. With an increased *α* it entails a greater influence of the covariance from *t* − 1 in sampling the covariance at *t*. *α* = 0.5 is reasonable given highly correlated BOLD time series. An *α* = 0 is more to be expected when time series are less correlated. 10,000 time points were sampled for each of the three different settings of the autocorrelation parameter. See also S2 Appendix for a justification of the parameter settings chosen here based on empirical fMRI data.

**Fig 5:**
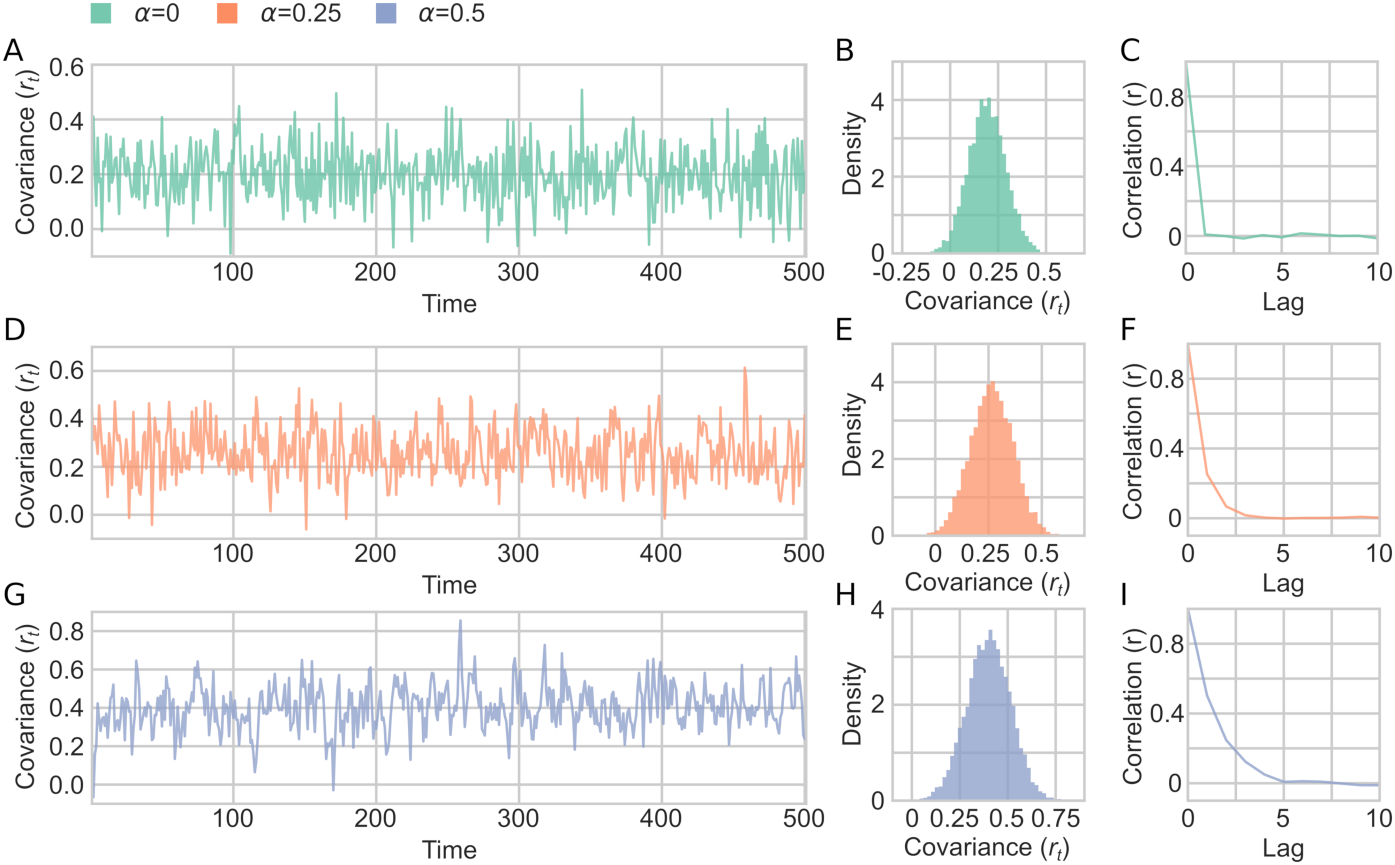
A sample of fluctuating covariance generated in Simulation 2. (A-C) *α* = 0 and *σ*_*r*_ = 0.1. (A) An example of *r*_*t*_ fluctuating over time, showing only first 500 time points shown for illustration purposes. (B) Distribution of the fluctuating covariance parameter (*r*_*t*_) (C) Autocorrelation of *r*_*t*_ for 10 lags. (D-F) Same as A-C but with *α* = 0.25. (G-I) Same as A-C but with *α* = 0.5.

Simulation 2 was run with 9 different simulation parameter combinations: three different values of *α* and three different values of *σ*_*r*_. A sample of time series generated with the model using different settings for the autocorrelation parameter *α* is shown in Fig 5A, 5D and 5G. Due to the varying degree of autocorrelation, the mean covariance for time series changes as a function of *α*, but *r*_*t*_ still depicts a Gaussian distribution (Fig 5B, 5E and 5H). The degree of crosscorrelation between the two time series followed the specified *α* parameter for the autocorrelation of the covariances (Fig 5C, 5F and 5I).

The results from Simulation 2 are shown in Tables 1–3 (for *σ*_*r*_ = 0.1) and Tables A-F in S3 Appendix (for *σ*_*r*_ = 0.08 and 0.12). The JC method had the lowest WAIC score for all settings of *α*, followed by the SD method. The MTD method came in third place for all but one parameter configurations. All WAIC values, their standard error and Δ WAIC scores are shown in Tables 1–3.

**Table 1:** Results of Simulation 2 where *α* = 0.0 and $\sigma_{r_{t}}$ = 0.1. Tables shows WAIC, WAIC standard error, and difference in WAIC from the best performing method. A lower WAIC indicates a better fit.

Model	WAIC	WAIC SE	Δ WAIC
JC	28103.6	142.963	0
SD	28104.3	143.047	0.687371
TD	28200.4	143.872	96.7158
TSW	28201.9	143.896	98.2981
SW	28205.8	143.956	102.192

**Table 2:** Results of Simulation 2 where *α* = 0.25 and $\sigma_{r_{t}}$ = 0.1. Tables shows WAIC, WAIC standard error, and difference in WAIC from the best performing method. A lower WAIC indicates a better fit.

Model	WAIC	WAIC SE	Δ WAIC
JC	28104.6	139.337	0
SD	28117.6	139.386	12.9322
TD	28168.1	139.483	63.5039
TSW	28181.8	139.501	77.1804
SW	28195.7	139.752	91.0829

**Table 3:** Results of Simulation 2 where *α* = 0.5 and $\sigma_{r_{t}}$ = 0.1. Tables shows WAIC, WAIC standard error, and difference in WAIC from the best performing method. A lower WAIC indicates a better fit.

Model	WAIC	WAIC SE	Δ WAIC
JC	28037.2	141.83	0
SD	28053.5	141.797	16.2988
TD	28120.2	141.398	82.9943
TSW	28148.7	141.628	111.464
SW	28201.2	142.018	163.961

**Fig 6:**
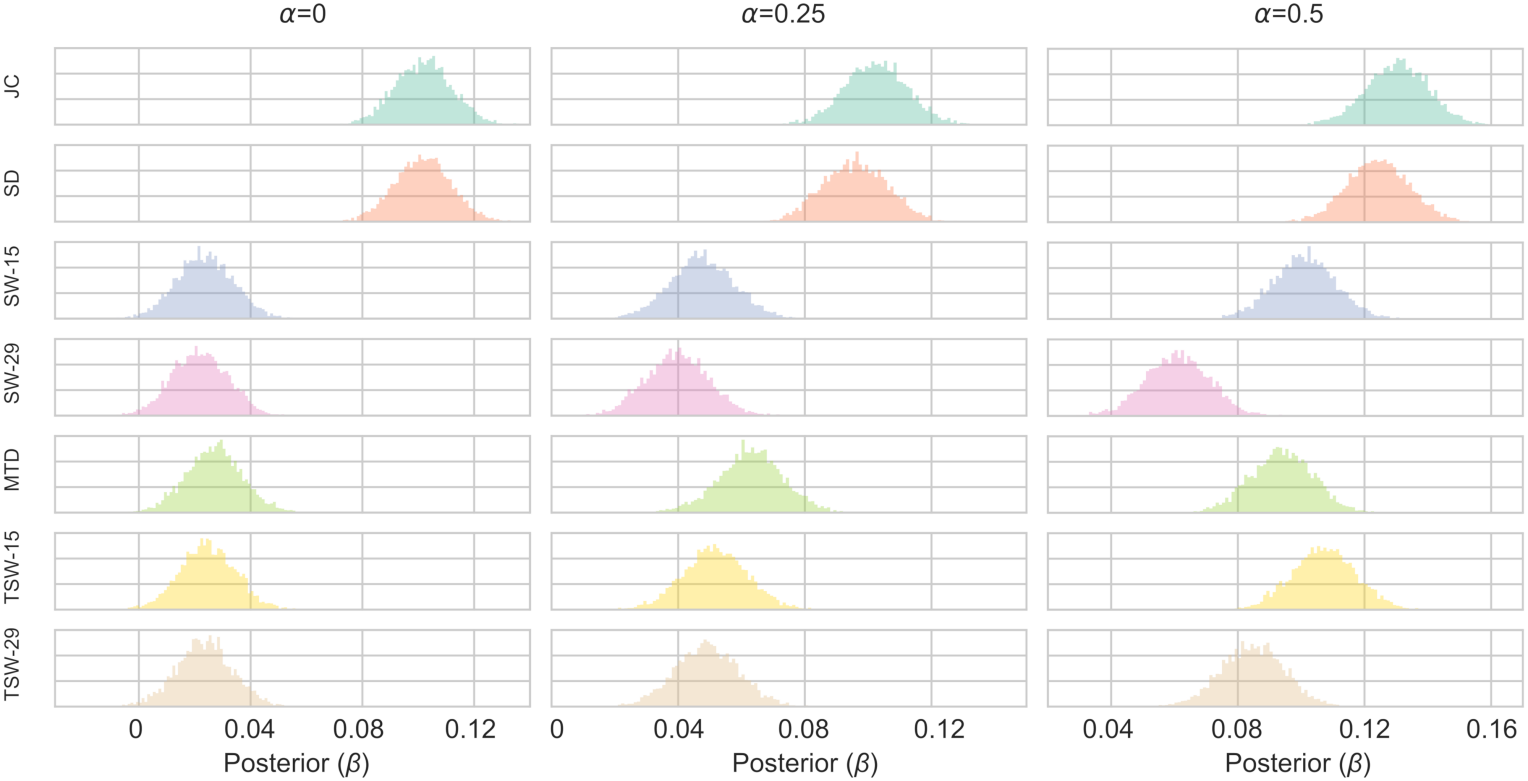
Posterior distributions of the *β* parameter of the Bayesian linear regression models in Simulation 2. The figure shows the results for varying values of the autocorrelation parameter (*α*) where the variance of the fluctuating covariance (*σ*_*r*_) is equal to 0.1. See S1 Appendix for other values of *σ*_*r*_. For each parameter configuration, a model was created for each TVC method. The TVC estimate was the independent variable estimating the fluctuating covariance (*r*_*t*_) between the two time series.

The posterior distribution of the *β* parameter for each of the TVC methods for all parameter choices are shown in Fig 6 when *σ*_*r*_ = 0.1 (for other values of *σ*_*r*_ see Figs A-B in S3 Appendix). Larger values in the *β* distribution for a method (i.e. correlating more with *r*_*t*_) conforms with the best fitting models (i.e. lower WAIC score). The SW-15, SW-29, TSW-15, TSW-29 and MTD methods performed equally poor when *α* = 0, and all improved as *α* increased. The MTD method improved the most as the *α* value increased, followed by the TSW-15 and SW-15 methods. SD and JC showed the best performance, with similar posterior distributions of *β*, although the JC was always slightly higher. There was little difference between the methods when changing the variance of the fluctuating covariance (*σ*_*r*_) (See S3 Appendix). The *β* values do however scale when *σ*_*r*_ changes. When *σ*_*r*_ is smaller, *β* values decrease due to there being more uncertainty when sampling each realization from similar distributions.

At times parts of the posterior distributions of the SW, TSW and MTD methods were below 0 to the extent that they would be not classed as “significant”. For example, these methods performed worst when *σ*_*r*_ = 0.08 and *α* = 0. Here the percentage of the posterior distribution above 0 was: SW-15: 80%, SW-29: 47%, TSW-15: 84%, TSW-29: 54%, MTD: 89%. The JC and SD methods always had the entire posterior distributions above 0.

In sum, the JC method, followed closely by the SD method, showed the best performance in terms of tracking a fluctuating covariance between two time series as performed in Simulation 2. The MTD method ranked in third place when there is a higher crosscorrelation between the time series present. The SW and TSW methods showed the worst performance, both in the WAIC score and posterior distributions of *β*.

### Simulation 3

The aim of Simulation 3 was to examine the behaviour of different TVC methods when there were non-stationarities present in the data. A typical scenario when this will occur is in a TVC analysis in task fMRI. Simulation 3 is identical in structure to Simulation 2 apart from the following two changes: (1) A non-stationarity, aimed to mimic the occurrence of an event related haemodynamic response function (HRF). Specifically *μ*, which was set to 0 for both time series in Simulation 2, received a different value at each *t* (see next paragraph). (2) *σ*_*r*_ was set to 0.1 instead of varying across multiple values. This is because Simulation 2 showed no large differences when varying *σ*_*r*_.

**Fig 7:**
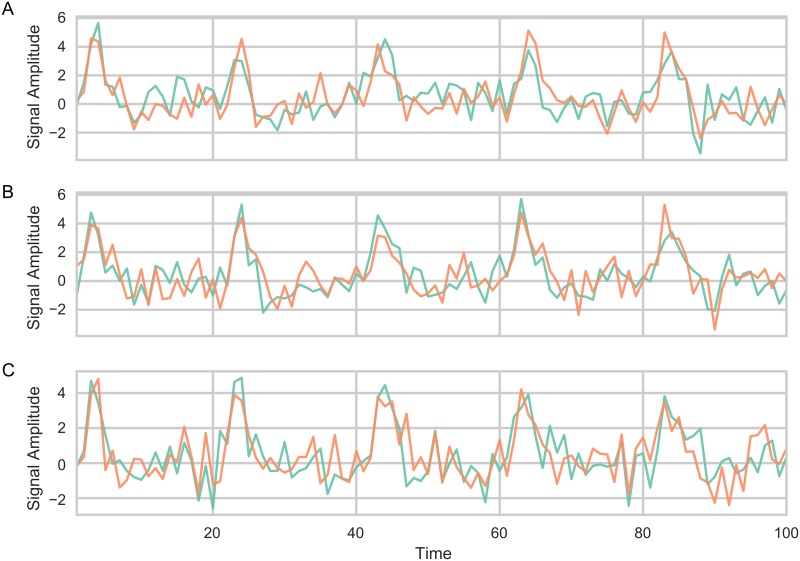
Examples of time series used in Simulation 3 where the mean of the time series is sampled from a time series that included a train of simulated event related HRF fMRI responses (spaced apart every 20 time points). Only the first 100 time points are shown for illustration purposes. (A) *α* = 0, (B) *α* = 0.25, (C) *α* = 0.5.

*μ*_*t*_ was set, for both time series, according to the value of a simulated HRF, that was twenty time points in length and repeated throughout the simulation. The HRF was simulated, with a TR of 2, using the canonical HRF function as implemented in SPM12 using the default parameters [40]. This HRF, which has a length of 17 time points, was padded with an additional 3 zeros. The amplitude of the normalized HRF was multiplied by 10 to have a high amplitude fluctuations compared to the rest of the data. *μ*_*t*_ is thus the padded HRF repeated throughout the entire simulated time series. This represents a time series that includes 250 “trials” that each lasts 40 seconds. This simulation helps illustrate how well TVC methods could be implemented in task based fMRI. Examples of the time series generated using different autocorrelation are shown in Fig 7.

**Fig 8:**
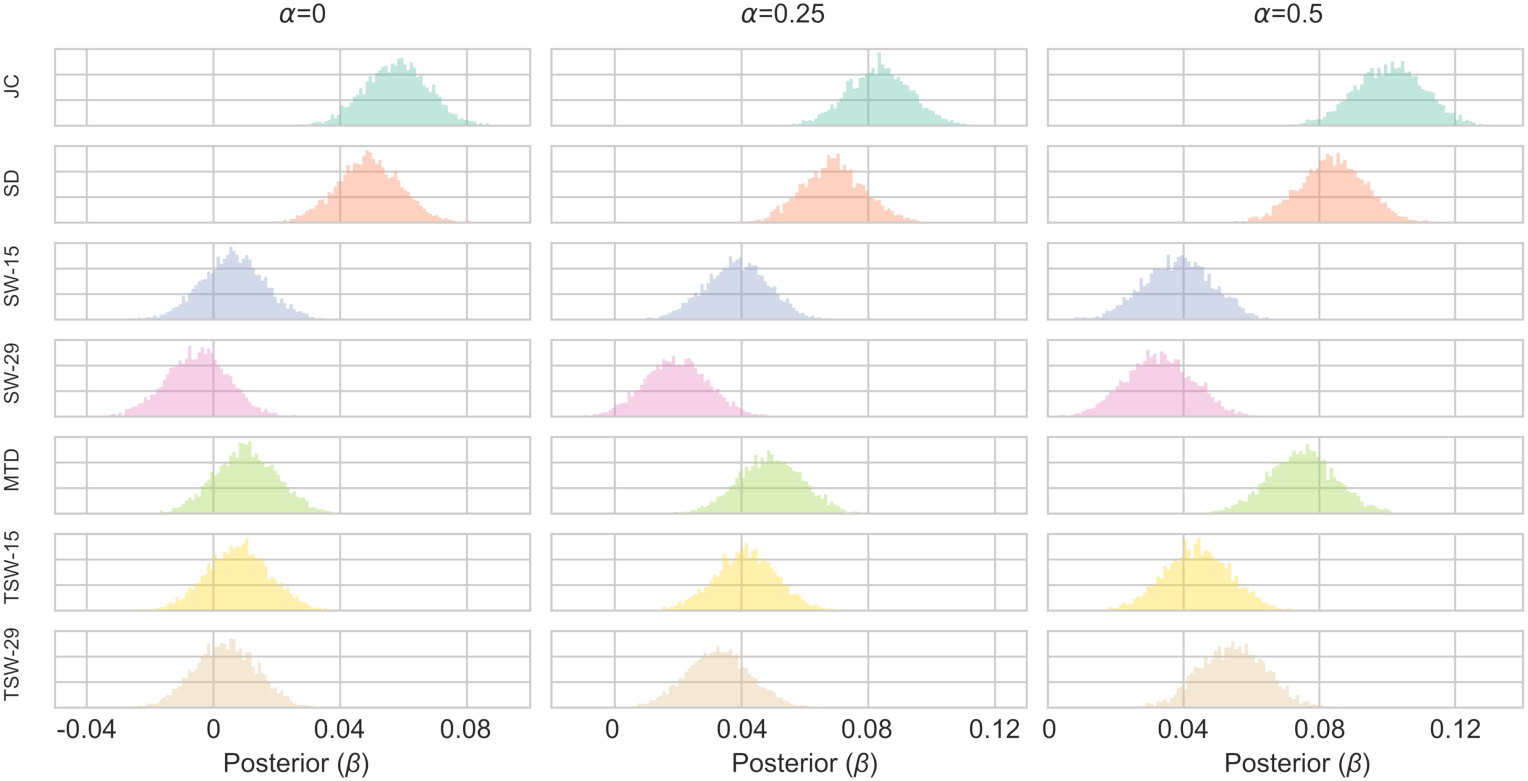
Posterior distributions of the *β* parameter of the Bayesian linear regression models in Simulation 3. Fig. shows a 1x3 grid with the varying values of the autocorrelation (*α*). For each parameter configuration, a model was created for each TVC method. The TVC estimate was the independent variable estimating the fluctuating covariance (*r*_*t*_) between the two time series.

The results from Simulation 3 are shown in Fig 8 (posterior distributions of *β*) and Tables 4–6 (model fit) which evaluated each TVC’s method performance at tracking the fluctuating covariance (*r*_*t*_). Results were similar with Simulation 2. In the case when the autocorrelation of the covariance was 0, the SW, TSW and MTD methods performed quite poorly, but again all improved to varying degrees as this increased. The longer windows (SW-29 and TSW-29) methods were generally the worst method, followed by shorter sliding window methods (SW-15 and TSW-15). The MTD method came in third place. The JC method has the best performance, followed closely by the SD method, in all parameter conditions. When *α* = 0, some methods had only portions of their posterior distribution above 0 (SW-15: 73%, SW:-29: 30%, TSW-15: 78%, TSW-29: 65%, MTD: 84%). The JC and SD methods had 100% of their distributions above 0 for all parameter conditions.

**Table 4:** Results of Simulation 3 where *α* = 0.0. Tables shows WAIC, WAIC standard error, and difference in WAIC from the best performing method. A lower WAIC indicates a better fit.

Model	WAIC	WAIC SE	Δ WAIC
JC	28175.3	142.323	0
SD	28184	142.346	8.68954
TD	28207.1	142.591	31.7657
TSW	28207.5	142.661	32.2356
SW	28207.6	142.653	32.2856

**Table 5:** Results of Simulation 3 where *α* = 0.25. Tables shows WAIC, WAIC standard error, and difference in WAIC from the best performing method. A lower WAIC indicates a better fit.

Model	WAIC	WAIC SE	Δ WAIC
JC	28138.3	142.606	0
SD	28160.5	142.749	22.2079
TD	28184.1	143.06	45.8161
TSW	28190.6	143.249	52.3508
SW	28202	143.265	63.7404

**Table 6:** Results of Simulation 3 where *α* = 0.5. Tables shows WAIC, WAIC standard error, and difference in WAIC from the best performing method. A lower WAIC indicates a better fit.

Model	WAIC	WAIC SE	Δ WAIC
JC	28106.1	139.883	0
SD	28136.7	139.769	30.6289
TD	28150.5	139.587	44.4145
TSW	28177.2	139.546	71.1701
SW	28203	139.717	96.9538

In sum, the results from Simulations 2 and 3 suggests that the JC method has the best performance in terms of detecting fluctuations in covariance compared to the other four TVC methods. This result also holds when a non-stationary event related haemodynamic response was added to the mean of the time series.

### Simulation 4

Simulation 4 aimed to test how sensitive different TVC methods are to large and sudden changes in covariance (i.e. changes in “brain state”) that previously have been postulated to exist in fMRI data (e.g. [11, 15, 17]). We here start in a similar fashion as we did in Simulation 2 where samples for the two time series are drawn from a multivariate Gaussian distribution

$$\begin{array}{c} X_{t}∼N\left(\mu_{t},\Sigma_{t}\right) \end{array}$$ (15)

$$\begin{array}{c} \Sigma_{t}=(\begin{array}{cc} \sigma & r_{t}\\ r_{t} & \sigma \end{array}) \end{array}$$ (16)

Similar to simulation 2, we set *μ*_*t*_ = 0 and *σ* = 1. The covariance parameter *r*_*t*_ was sampled from a Gaussian distribution where the mean was shifted

$$\begin{array}{c} r_{t}∼N\left(\mu_{{\text{state}}_{t}},\sigma_{r}\right) \end{array}$$ (17)

and where *σ*_*r*_ = 1. At each state transition, $\mu_{{\text{state}}_{t}}$ was randomly chosen from a set *M* (*M* = {0.2, 0.6}). The duration of each state was randomly sampled from *L*. Two different scenarios for state transitions were simulated. In the fast transition condition *L* = {2, 3, 4, 5, 6} and in the slow transition condition *L* = {20, 30, 40, 50, 60}. These values correspond to the number of time points a “state” lasts. Beginning at *t* = 1, $\mu_{{\text{state}}_{t}}$ to $\mu_{{\text{state}}_{t+l}}$ was randomly sampled from *M* where *l* was sampled from *L*. This procedure was continued until *X*_*t*_ was 10,000 samples long.

**Fig 9:**
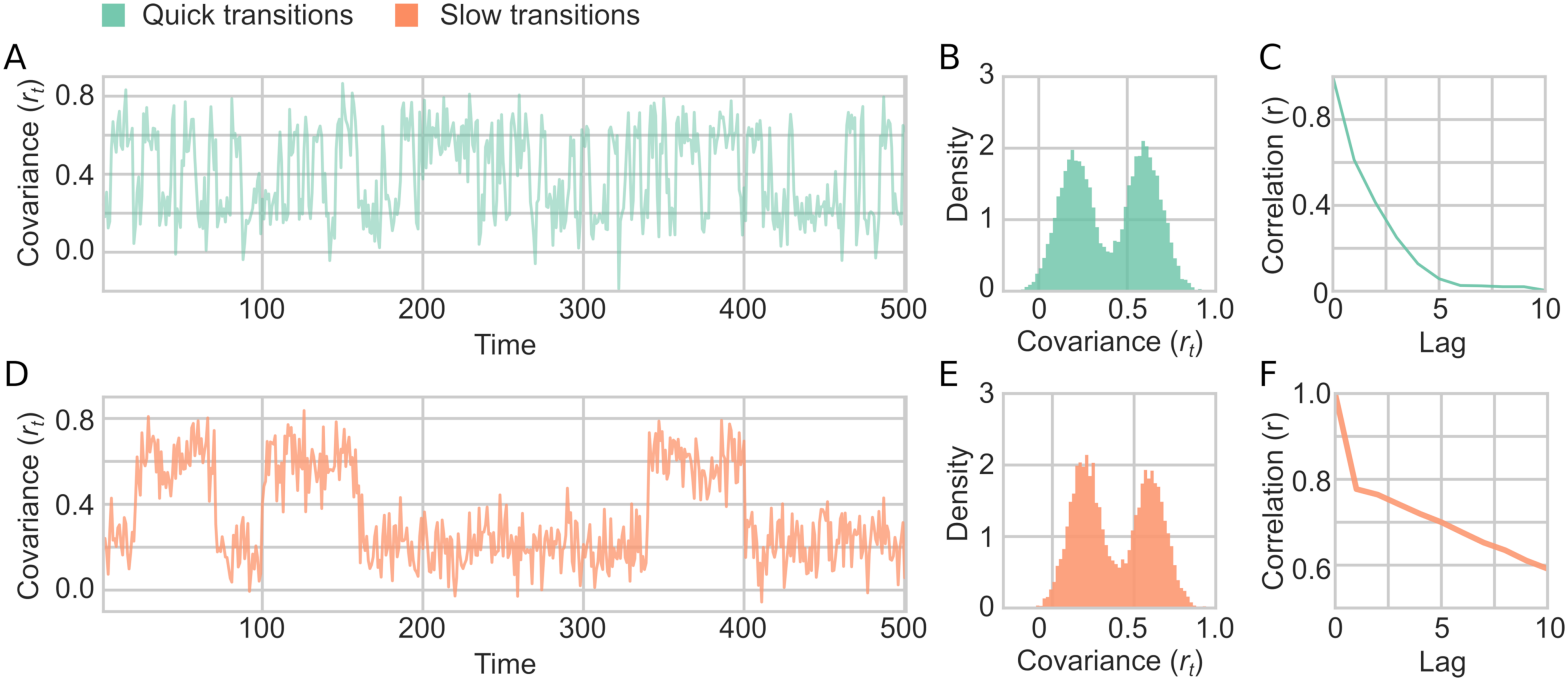
A sample of fluctuating covariance generated in Simulation 4. (A-C) Quick state transitions (between 2-6 time points long). (A) An example of *r*_*t*_ fluctuating over time, showing only first 500 time points shown for illustration purposes. (B) Distribution of the fluctuating covariance parameter (*r*_*t*_) (C) Autocorrelation of *r*_*t*_ for 10 lags. (D-F) Same as A-C but with the long state transitions (between 20-60 time points long).

These choices for brain state changes provide time scales of state transitions between 40-120 seconds (slow condition) or 4-12 seconds (fast condition) in simulated fMRI data with a TR of 2 (Fig 9A and 9D). The statistical model for evaluating the different TVC methods performance was the same as Simulation 2 and 3. A summary of data generated in Simulation 4 is shown in Fig 9.

**Fig 10:**
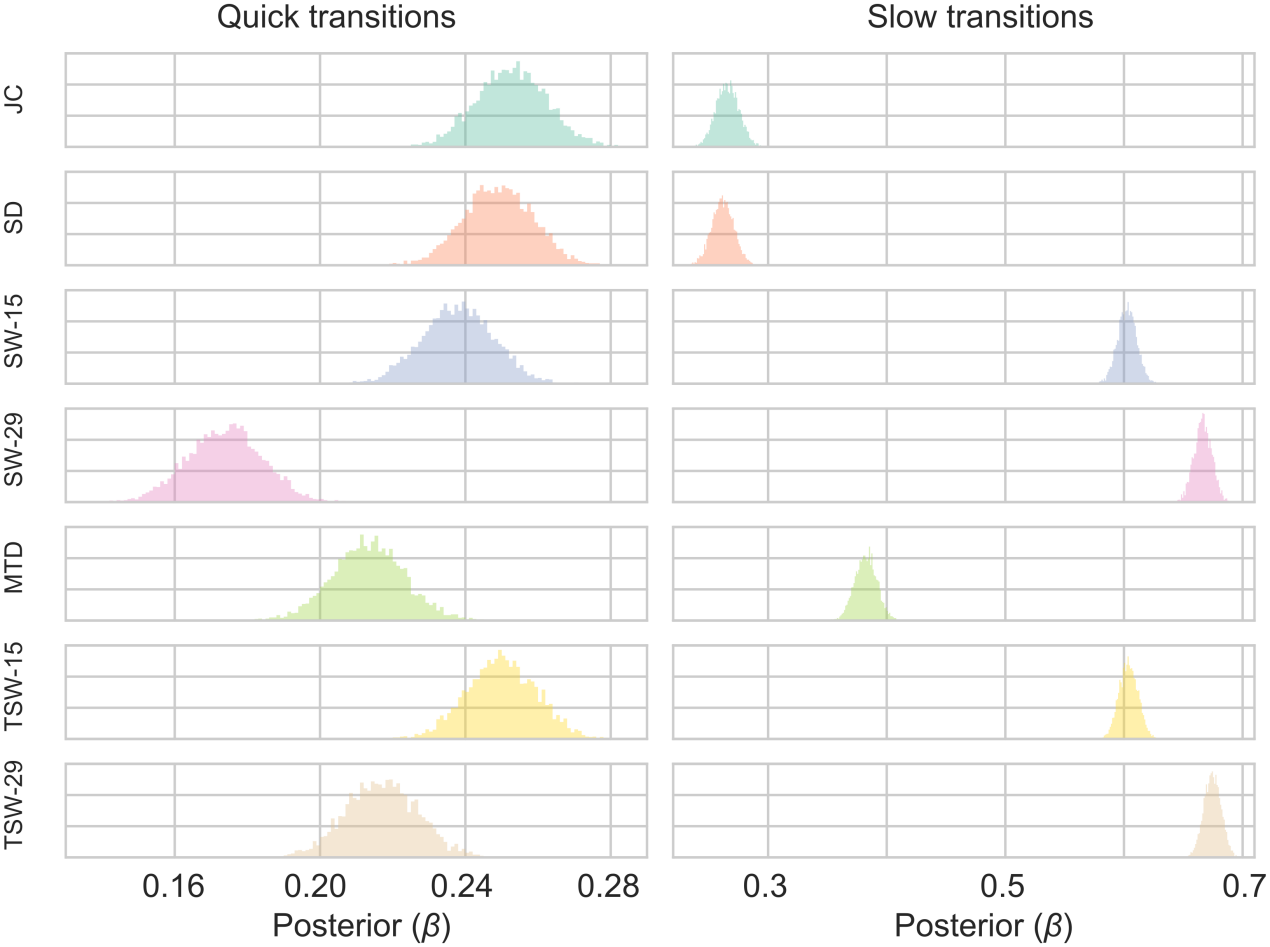
Posterior distributions of the *β* parameter of the Bayesian linear regression models in Simulation 4. Fig. shows a 1x2 grid with the varying values of the state length. For each parameter configuration, a model was created for each TVC method. The TVC estimate was the independent variable estimating the fluctuating covariance (*r*_*t*_) between the two time series.

The results from Simulation 4 are shown in Fig 10 and Tables 7 and 8. In the quick transition condition, the JC and the SD showed the best performance for both the WAIC scores and the posterior distribution of *β* (Fig 10A; Table 7). This was followed by the SW-15 and TSW-15 methods. In the slow transition condition the two sliding window methods outperformed the other methods (Fig 10B; Table 8), with the longer windows (TSW-29 and SW-29) being outperforming the shorter windows. The JC and SD methods perform similarly for both conditions. Thus, when there are shifts in covariance that occur relatively slowly, the sliding window methods are sensitive at tracking these changes. All methods had 100% of their posterior distributions above 0.

**Table 7:** Results of Simulation 4 where state length = {2,3,4,5,6}. Tables shows WAIC, WAIC standard error, and difference in WAIC from the best performing method. A lower WAIC indicates a better fit.

Model	WAIC	WAIC SE	Δ WAIC
JC	27548.3	92.0207	0
SD	27571.1	92.7548	22.8124
TD	27741.6	93.5845	193.243
TSW	27749.5	93.3275	201.18
SW	28072.5	87.9986	524.197

**Table 8:** Results of Simulation 4 where state length = {20,30,40,50,60}. Tables shows WAIC, WAIC standard error, and difference in WAIC from the best performing method. A lower WAIC indicates a better fit.

Model	WAIC	WAIC SE	Δ WAIC
TSW	21730.5	144.261	0
SW	22796.5	139.927	1065.97
TD	26630.8	106.131	4900.32
JC	27478.9	92.087	5748.42
SD	27503.1	93.3021	5772.6

## Discussion

In this study we have developed four simulations to test the performance of different proposed time-varying connectivity methods. The first simulation showed which methods yield similar connectivity time series. Notably, all methods correlated positively with each other, but to a varying degree. The second simulation generated data in which the autocorrelated covariance between simulated time series varied in time. In this case, the JC method, followed closely by the SD method, showed the best performance. In the third simulation, the generated time series contained a non-stationary mean related to haemodynamic responses. Again, our simulations suggested that the JC method performed best. The fourth simulation included nonlinear shifts in covariance (in an attempt to simulate brain state shifts). When the states changes were quick, the JC method performed best. When the state changes were slow, the TSW (followed by the SW) performed best.

In a previous simulation that evaluated the sliding window method, the sensitivity of the SW and TSW methods was found to be good at detecting state shifts [41]. Here, at least when the transitions are slow, we found similar results. The sliding window methods is optimal if there are slow state changes. However it is unclear if “state changes” are the best yardstick for time-varying connectivity. In particular, non-stationarities in time-varying connectivity have been attributed to spurious sources such as movement [12]. Given the unknowns of the “true” connectivity, methods which are robust over conditions are more likely the safer options—in this case the JC or SD method performed similarly in both conditions. However, as mentioned in the methods section, the SD method tested here is the bivariate version of the method and not the multivariate version previously proposed in [8] (see also S1 Appendix for more the relationship between these methods).

Overall the jackknife correlation method performed the best across all simulations. We have shown it to be robust to numerous changes in parameters. However, the JC method is not without some considerations. First, it introduces variance compression that reduces the absolute variance, while preserving the relative variance within the time series. This variance compression also scales with the length of the time series. The consequence of this is that direct comparisons of the TVC variance between cohorts/conditions become hard to interpret as time-varying fluctuations, especially when the length of the data varies. However, this is the case for most methods and it should be remembered that the variance is proportional to the static functional connectivity [7, 9, 10]. Simply put, the JC method (like all other methods) should not be used for a direct contrast of the variance of TVC time series. Second, the JC method sensitivity means that noise will be carried over per time point instead of being smeared out over multiple time points. This is actually beneficial as it allows for further processing steps to be applied that aim to remove any remaining noise (e.g. motion) which cannot be done when the noise has been smeared across the connectivity time series (e.g. in windowed methods).

The simulations and results presented in this study should not be taken as an exhaustive and complete assessment of all aspects of a given method to conduct TVC. Rather, the four simulations described here represents a subset of possible scenarios in terms of different methodological characteristics that might be of interest. The current four simulations are marked *tvc_benchmarker* simulation routine V1.0. If modifications or additional scenarios are considered to be improvements to the current simulations, these will get an updated version number. Many additional simulations could be conceived on top of this original routine. For example, one could include multiple time series, adding movement type artifacts, adding frequency relevant characteristics, a stationary global signal etc. These have not been included here, as the focus in these simulations was to primarily assess tracking of a fluctuating covariance. Input from researchers about appropriate additions to the simulations is welcome.

We encourage researchers designing TVC methods to benchmark their own results with *tvc_benchmarker* (www.github.com/wiheto/tvc_benchmarker). Researchers need only to write a Python function for their method and use it as an input for tvc_benchmarker.run_simulations() and their method will be compared to the TVC methods presented in this paper (see online documentation). Functions can then be submitted through the function tvc_benchmarker.send_method(). All valid methods submitted will be released in summaries of the submitted benchmarked results so that researchers can contrast the performance of different methodologies.

## Supporting information

S1 Appendix Bivariate vs. multivariate SD methods. (PDF)

S2 Appendix A justification of the assumptions regarding model parameter choices made in the simulations. (PDF)

S3 Appendix Varying the variance of *r*_*t*_ in Simulation 2. (PDF)
