# Supplementary material for: Simulations to benchmark time-varying connectivity methods for fMRI
Source: PLoS Comput Biol. 2018 May 29;14(5):e1006196. doi: 10.1371/journal.pcbi.1006196 (PMC5993323; doi:10.1371/journal.pcbi.1006196)
Supplement: S1 Appendix — (PDF) [file pcbi.1006196.s001.pdf]

S1 Appendix: bivariate vs. multivariate SD methods.

The SD method constructs a weight vector for each time point and then uses this weight vector in a weighted Pearson correlation to estimate the ongoing fluctuations (See @Thompson_teneto; @Thompson2018). The weight vector for time point t is constructed by calculating the distance of the spatial dimensions between t and every other time point. In the original presentation of the SD method [@Thompson_teneto] the idea was to use all spatial dimensions to assist the estimation. By using all spatial dimensions the *global configuration* effects the weighting which is an assumption of the method. For example, if every ROI increases in their signal amplitude (e.g. due to an artifact) the weighting would be different than if only two ROIs increase in their amplitude. This is the *multivariate SD* method. One benefit of the multivariate SD case is that it

In the main text, only the *bivariate SD* method is considered since, in this version of TVC_benchmarker, only two time series are generated. Now it is only the two time series themselves, with no additional time series, that generates the weight vector. This leads to an important question whether any result drawn on the bivariate SD method is related to the multivariate SD method.

The MSC fMRI data and the random subject/session that is outlined in S2 Appendix (MSC10/func07). Both the bivariate and multivariate spatial distance method was applied to all 38,503 edges. S1 Appendix, Fig. A shows the distribution of the correlation values for all 38,503 edges. The mean Spearman correlation value is 0.76 (min: 0.53; max: 0.91).

While the bivariate and multivariate methods do show some difference and this should be tested in future versions of `TVC_benchmarker`, we can see that the bivariate version of the SD method is, at the very least, an adequate approximation.

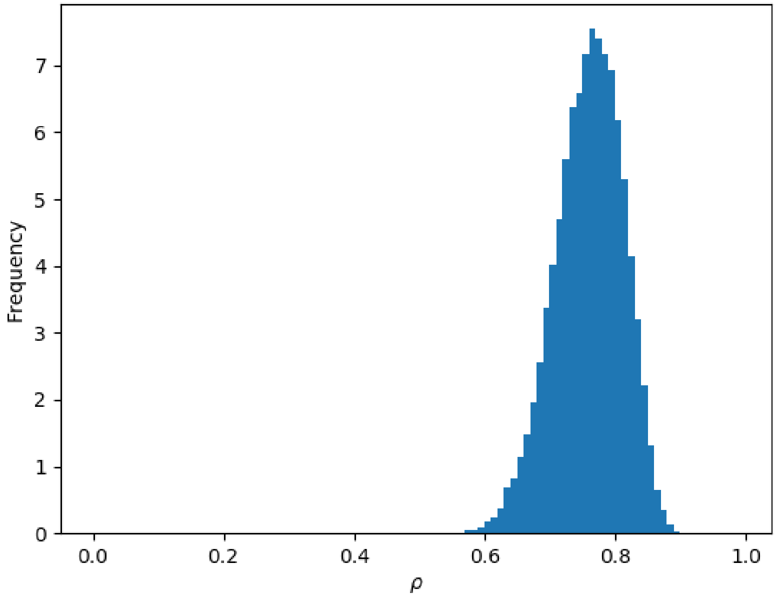

Figure A: Distribution of Spearman ρ values between the bivariate and multivariate SD measures for 38,503 edges.
