# Supplementary material for: Simulations to benchmark time-varying connectivity methods for fMRI
Source: PLoS Comput Biol. 2018 May 29;14(5):e1006196. doi: 10.1371/journal.pcbi.1006196 (PMC5993323; doi:10.1371/journal.pcbi.1006196)
Supplement: S2 Appendix — (PDF) [file pcbi.1006196.s002.pdf]

S2 Appendix: A justification of the assumptions regarding model parameter choices made in the simulations.

In simulation routine V1.0 of tvc_benchmarker, we wanted the simulations to either be justified based on the properties of fMRI data or, in the case of Simulation 4, reflect what dynamics are hypothesized to exist in fMRI data.

Since the properties of the simulations are determined by their parameters, we justify these parameters in three different ways. First, we show that some parameters have no substantial effect on the result. Second, some are justified from previous findings/hypothesis in the literature. Third, by use resting state fMRI data to justify the assumptions.

fMRI data used

The midnight scanning club (MSC) data [1] is used to justify the assumptions. The dataset contains ten subjects with ten sessions of resting state fMRI. This data was obtained from the OpenfMRI database (accession number: ds000224). The preprocessed volumetric data was used. To reduce the dimensions of the data, 278 spherical regions of interest were defined (5mm radius) with their the centre of mass defined by the Shen et al 2013 atlas [2] (using the Talairach coordinates). Subject MSC08 is often reported as an outlier, which has previously been noted in Gordon et al where they noted this subject had poor data quality due to subject head movement and sleep [1]. When a single subject and and session is shown (for illustration purposes), subject MSC10 and session 7 was used. This subject and session was selected at random. When specific ROIs were selected to be shown, these were randomly generated (from subject MSC10, session 7).

See github.com/wiheto/tvc_benchmarker_assumptions/1.0/assumptions.ipynb for the Jupyter notebook to run all the analysis from start to finish.

Simulation 1 - summary of assumptions

Information

Aim: See how well the estimates from different DFC methods correlate with each other.

Evaluation: Spearman correlation between functional connectivity time series computed by the five DFC methods.

n samples: 10,000

Random seed: 2017

Assumptions

Distributions: Gaussian, multivariate Gaussian

μ *(mean of each time series)*: 0

σ *(variance of time series)*: 1

r_t *(covariance of time series)*: 0.5

α *(autocorrelation of the time series)*: 0.8

Simulation 2 - summary of assumptions

Information

Aim: To investigate how well the different DFC methods correlate with a fluctuating covariance parameter.

Evaluation: Bayesian linear regressions between the estimates of the DFC methods and r_t (evaluated by comparing WAIC scores and posteriors of β).

n samples: 10,000

Random seed: 2017

Assumptions

Distributions: Gaussian, multivariate Gaussian

μ *(mean of each time series)*: 0

σ *(variance of time series)*: 1

μ_r *(mean of fluctuating covariance)*: {0.1, 0.2}

σ_r *(variance of fluctuating covariance)*: {0.08, 0.1, 0.125}

α *(autocorrelation of the fluctuating covariance)*: {0, 0.25, 0.5}

Simulation 3 - summary of assumptions

Information

Aim: To investigate how well different DFC methods perform when the fluctuating covariance parameter contains a non-stationary mean to simulate a HRF.

Evaluation: Bayesian linear regressions between the estimates of the DFC methods and r_t (evaluated by comparing WAIC scores and posteriors of β).

n samples: 10,000

Random seed: 2017

TR: 2

Assumptions

Distributions: Gaussian, multivariate Gaussian

μ A scaled HRF.

σ *(variance of time series)*: 1

μ_r *(mean of fluctuating covariance)*: 0.2

σ_r *(variance of fluctuating covariance)*: 0.1

α *(autocorrelation of the fluctuating covariance)*: $\{0, 0.25, 0.5\}$

Trial length (samples): 20

HRF scale: 10

Simulation 4 - summary of assumptions

Information

Aim: To investigate the performance of DFC methods when there is a fluctuating covariance parameter that nonlinearly shifts between covariance.

Evaluation: Bayesian linear regressions between the estimates of the DFC methods and r_t (evaluated by comparing WAIC scores and posteriors of β).

n samples: 10,000

Random seed: 2017

Assumptions

Distributions: Gaussian, multivariate Gaussian

μ *(mean of each time series)*: 0

σ *(variance of time series)*: 1

r_μ *(average covariance in different states)*: 0.2, 0.6

r_σ *(variance of covariance in different states)* : 0.1

State length: Fast Condition: {2,3,4,5,6}; Slow condition: {20,30,40,50,60}

Justifying the assumptions

Mean and variance of the time series (μ, σ)

μ and σ for all simulations were set to 0 and 1, with the exception of μ in simulation 3. As long as the time series have a stationary mean and variance, then these parameters can be set to anything and have no effect on the overall result. Simulation 3 tests how methods deal with the non-stationartity of μ.

Autocorrelation and crosscorrelation assumptions (α)

Autocorrelation of the time series (Simulation 1).

The auto-correlation (α) is an important parameter for simulation 1. In Appendix S2, Fig. A, we can see the average auto-correlation for a single subject between 0 and 10 lags. The average and standard deviation for each subjects and session is shown in Appendix S2, Fig. B. Excluding the sessions from the subject known to be noisy (MSC08), there were only a few sessions with average auto-correlation values outside the range of 0.70-0.85. Most subject averages were close to 0.80. Accordingly, α in simulation 1 was set to 0.80.

Autocorrelation of r_t (Simulation 2 and 3)

The autocorrelation of r_t is an important parameter for simulation 2 and 3, where the parameter α now refers to the autocorrelation of the covariance parameter r_t. The expected crosscorrelation will be different for different values of static functional connectivity. Thus, we binned each edge based on its static connectivity bins (each bin was placed between -1 and 1 in spaces of 0.1). Panel A in Appendix S2, Fig. C shows the average crosscorrelation of the example subject and session for 10 lags for each bin. The general pattern is that at lag 1, the average correlation tends towards zero compared to lag 0 (which is the static functional connectivity). The frequency of the number of edges across the different correlation values is at lag 0 (Panel B in Appendix S2, Fig. C) and lag 1 (Panel C in Appendix S2, Fig. C) shows that the majority of edges are within the range -0.5 and 0.5.

The average crosscorrelation at lag 1 looks very similar across different subjects and sessions (Appendix S2, Fig. D). When pooling all static functional connectivity values across subjects and sessions, the histogram (Appendix S2, Fig. E) shows that the pattern at a group level is similar to that shown in panel B Appendix S2, Fig. C. Thus, more edges correspond to a degree of connectivity that would be modeled with a lower α parameter. However, for edges with larger degree functional connectivity, a higher α is to be expected.

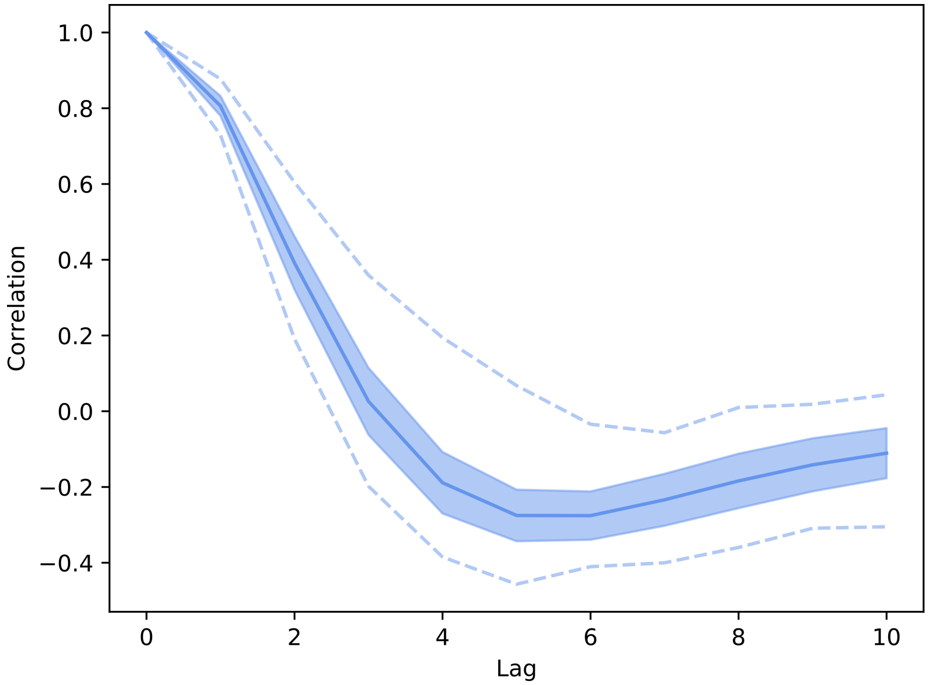

Figure A: Auto-correlation of the example subject and session for 10 lags. Averaged over all ROIs. Error bars show standard deviation. Dashed lines show the min and max.

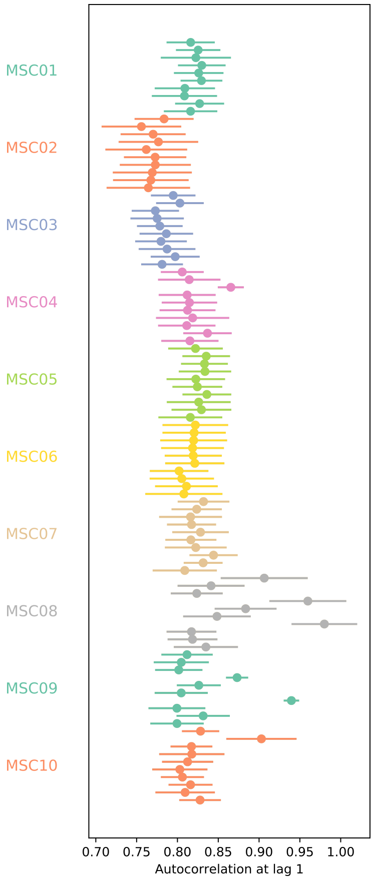

Figure B: Average auto-correlation at lag 1 for each subjects and sessions. Each dot represents a session (ordered from top to bottom). Each dot signifies the average autocorrelation at lag 1. Error bars show standard deviation.

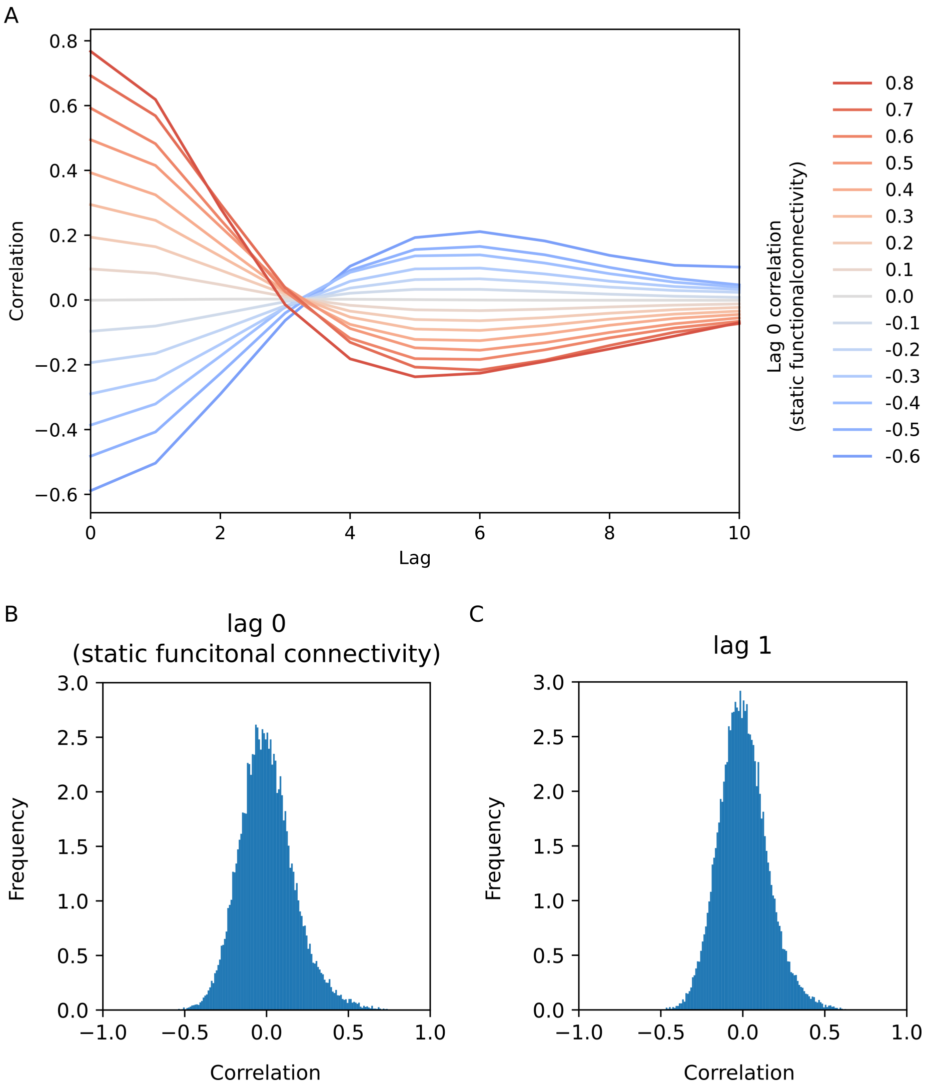

Figure C: Average crosscorrelation of the example subject and session for 10 lags. Averaged over all ROIs. Each coloured line represents a bin based on the correlation value at lag 0 (i.e. the static functional connectivity).

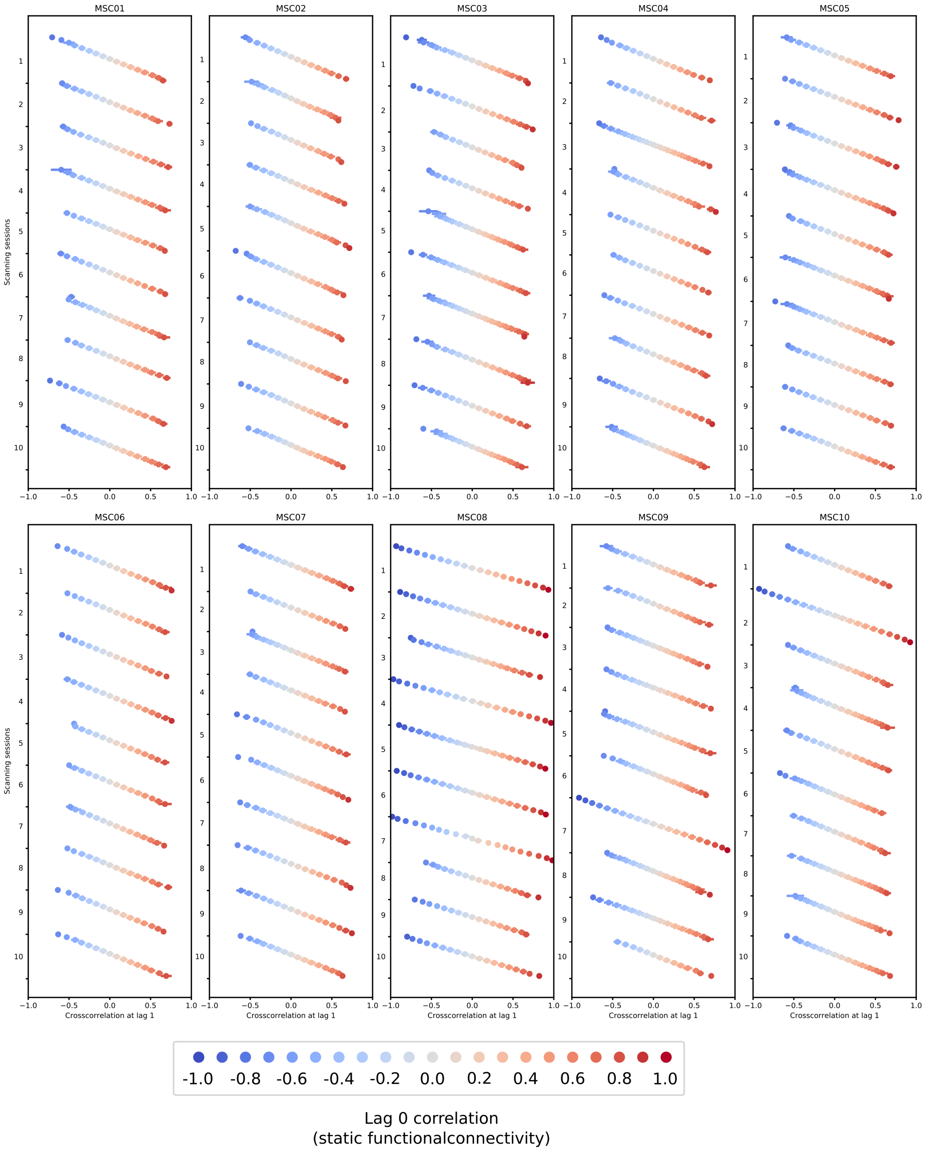

Figure D: Average crosscorrelation at lag 1 for all subjects and sessions. Each dot represent a bin based on the correlation value at lag 0 (i.e. static functional connectivity). Each dot signifies the average (across ROIs in the bin) crosscorrelation at lag 1. Error bars indicate standard deviation.

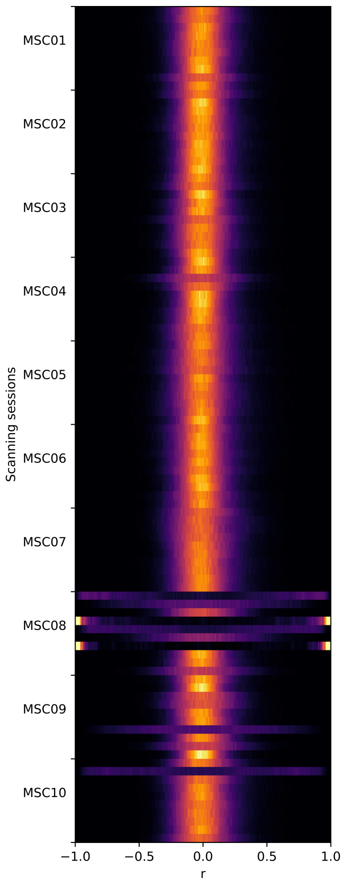

Figure E: The frequency of the static functional connectivity for each subject and session. Each row in the figure represents one session for a subject. Each row contains a normalized histogram of the connectivity values.

When choosing the α for simulation 2 and 3, we only considered positive autocorrelations and sampled from the values 0, 0.25, and 0.5 which cover most of the positive span of functional connectivity.

Covariance (μ_r, σ_r, r_t)

σ_r dictates how wide r_t is going to fluctuate over time. What the true value of σ_r is, is one of the key questions time-varying functional connectivity wants to answer. Making σ_r higher, entails that there is a larger parameter space that r_t gets sampled from which decreases the noise when estimating r_t. When the distribution that r_t is sampled from widens, there is a lower probability that the time series will be sampled similarly, meaning the inherent uncertainty of estimating single samples of r_t decreases when the variance of r_t increases. The effect of changing σ_r is that the β values will scale with it, but the relative difference of the β values between methods will remain similar. The σ_r parameter in is set to 0.08, 0.1 and 0.12 to illustrate this in simulation 2 to show that this has little effect other than a scaling effect of β (Figure 6). In simulation 3, only 0.1 was used for σ_r.

The average connectivity (μ_r) was set 0.2 in simulation 2 and 3. It can be seen in Appendix S2, Fig. E that both this value is reasonable positive connectivity value. When varying the α parameter in simulation 2 and 3, this also has an effect on the effective covariance (mean of r_t) between the two time series and this in increases as a function of α (Figure 5). It can be seen that this effective increase in covariance had little effect on the different methods (Figure 6, Figure 8).

As the mean r_t increases when α increases, it is possible that any change in a method's performance occurs due to the change in the mean of r_t and not α. To this end, we created an additional supplementary parameter routine in tvc_benchmarker (1.0sup). This simulation followed all the structure of simulation 2. α was set to 0.25. σ_r was set to 0.1. μ_r changed between 0.2 and 0.3. In Appendix S2, Fig. F we show the posterior distributions of the β values for the two different values of μ_r. Here it can be seen that there is a slight shift in the β values as μ_r increases, but no notable difference in the relative performance of the methods. This shows that the differences between the methods as α increases in Simulation 2 is due to α, not the mean of r_t changing due to α.

The r_t in simulation 4 switched between a very high connectivity "state" of 0.6 and lower, but positive, connectivity "state" of 0.2. These are plausible connectivity values (Appendix S2, Fig. E). See the section below on state change assumptions for more details.

While the parameters defining draws of r_t has been justified above on what is considered reasonable, μ_r and σ_r parameters were defined in such a way that

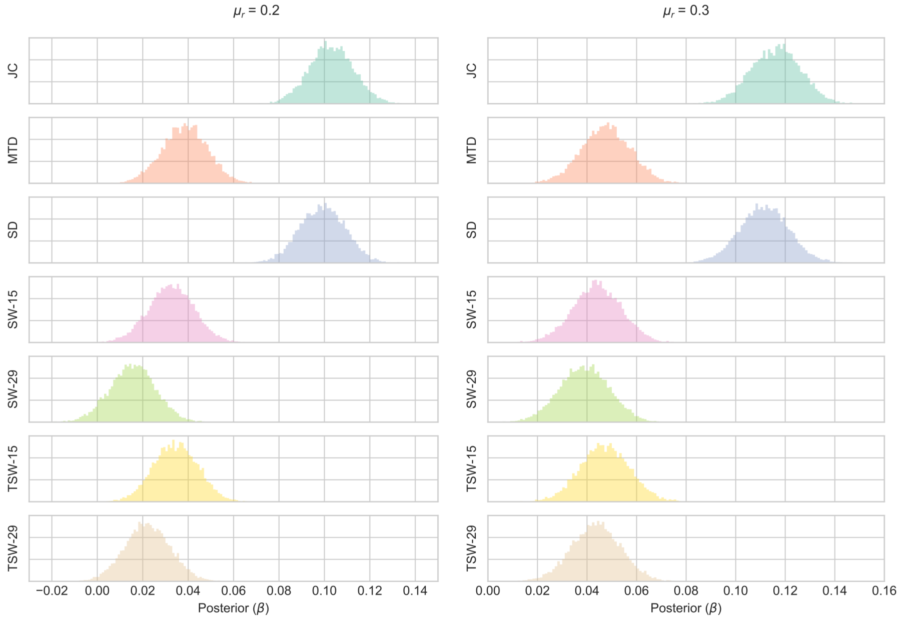

Figure F: Distribution of BOLD signal for each ROI in example subject and session. A. Histograms over each ROI showing the frequency of signal intensity. Each row functions as a histogram for a ROI. B. Three randomly selected ROIs from A are depicted as traditional histograms showing the same information as in A.

there was no instance when $r_t > 1$. This motivates using the range of of σ_r being between 0.08 and 0.12 instead of having a much larger variance.

Gaussian assumption

All simulated time series were sampled from a (multivariate) Gaussian distribution. The simulations can be improved by considering frequency specific information (see caveats below). The amplitude of the time series for ROIs in the example subject and session have a unimodal shape and relatively unskewed distributions (Panel A in Appendix S2, Fig. G). To illustrate this more clearly, panel B Appendix S2, Fig. G shows three randomly selected ROIs. When looking at all groups the average skewness was calculated (Appendix S2, Fig. H). Given the distributions are unimodal they too can at least be approximated with a Gaussian distribution. While not perfect, and still a simplification, a Gaussian distribution is a reasonable distribution to sample from.

The Gaussian distribution assumption for sampling r_t can also be motivated by [3] where the time-varying functional connectivity was generally unimodal and, with appropriate transformation, approximates to Gaussian.

Trial length and HRF scale

The scale of the HRF amounts to how much the standardized HRF has been scaled so that it creates a larger non-stationarity in mean of the simulated data in Simulation 3. This parameter was set to a high value to illustrate each method's ability to estimate connectivity when a non-stationarity mean is present in the data. There will be little effect when changing this, but if it was reduced it will eventually be identical to simulation 2. The non-stationarity introduced by the adding the HRF function to the data is shown in Figure 7 of the main text.

The HRF lasting 20 seconds with an assumed TR of 2 means that there are 40 seconds between trials. This is quite long for an event related fMRI experiment. If this was reduced, it would just entail that the HRF stacks due to overlap. This would have no effect on what the simulation is testing which is just how well the different methods can act upon non-stationarities. The TR was considered to be 2 seconds because many studies still use this TR despite lower TRs being available. However, this has no implications on the results.

Assumptions regarding shift in "brain state"

The duration of a state in the quick condition are on the approximate time scale found in [4], although slightly quicker (where the average state transition was 7 seconds). The longer transitions are based on the time scale explored in [5]. Note however that these two different time scales for states originate from

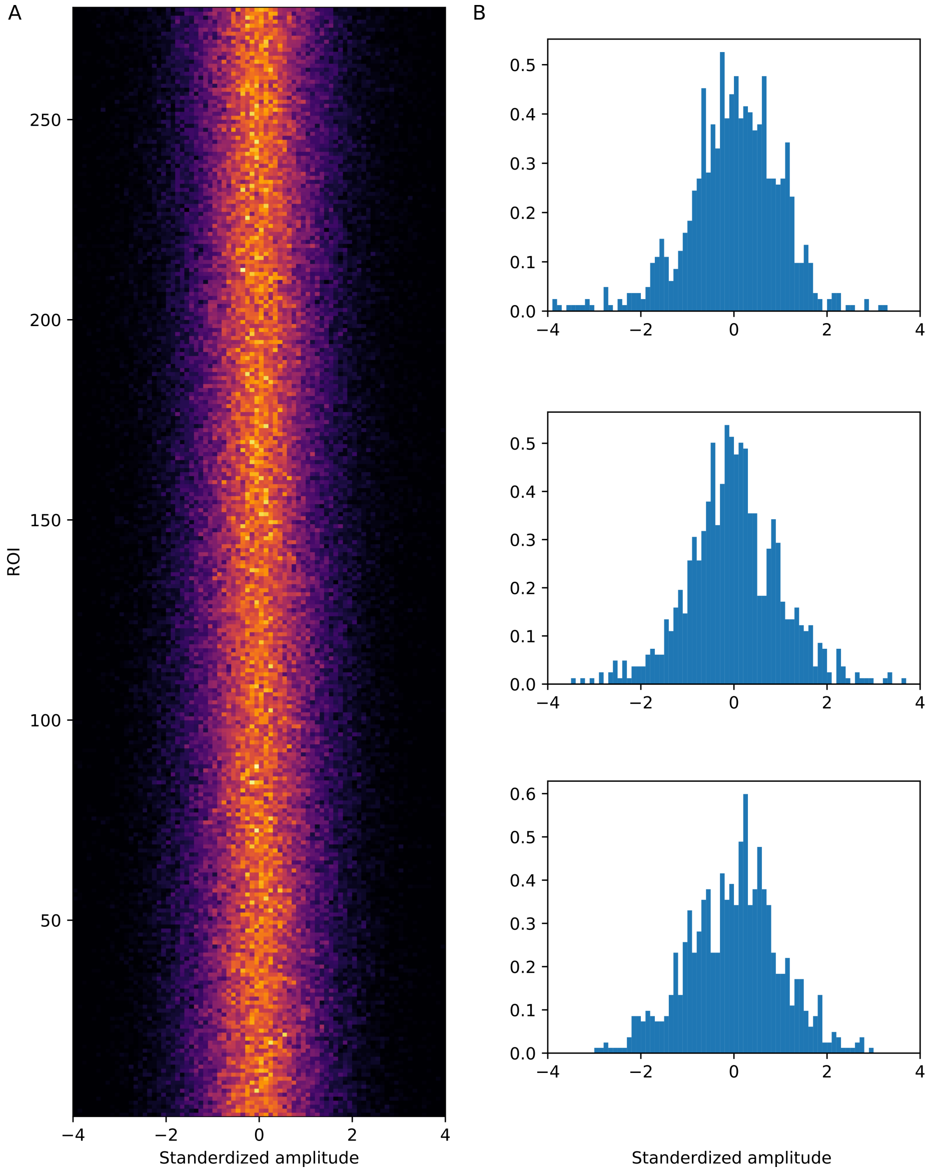

Figure G: Distribution of BOLD signal for each ROI in example subject and session. A. Histograms over each ROI showing the frequency of signal intensity. Each row functions as a histogram for a ROI. B. Three randomly selected ROIs from A are depicted as traditional histograms showing the same information as in A.

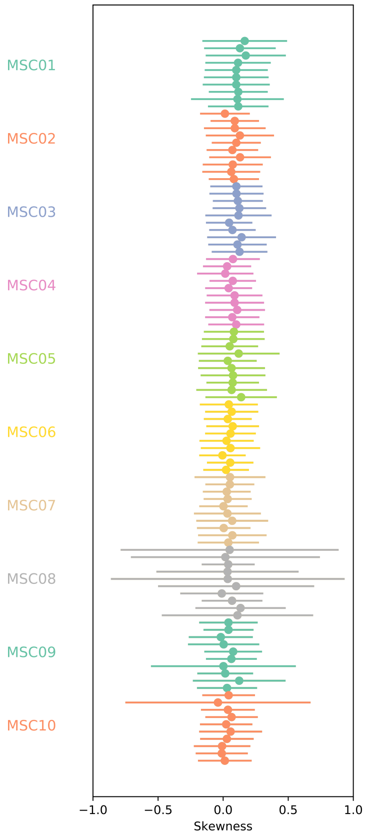

Figure H: Average skewness for each subject and session. Each dot represents a session. Each dot signifies the average skewness over all BOLD time series for ROIs. Error bars show one standard deviation

different time-varying functional connectivity methods. Detecting brain states has, to our knowledge, not been justified without using time-varying functional connectivity which makes the reasoning to justify this parameter somewhat circular. Simulation 4 was added primarily to evaluate how the different methods can capture dynamics that many researchers hypothesize time-varying functional connectivity to be, not necessarily how the data is.

Caveats

The current simulations do not take into account any frequency information of fMRI brain connectivity which is known to play a role in resting state fMRI. This means that any methods that utilize this aspect of the signal (e.g. phase) cannot be evaluated at the moment. The Gaussian assumption of the distributions can be improved upon by adding more fMRI specific noise. Further, drawing r_t from a Gaussian distribution was done to keep Gaussian assumptions throughout the paper instead of adding additional distribution assumptions for different aspects of the simulations. However, since r_t should always be bound between -1 and 1, a beta distribution could be a more appropriate in subsequent versions of the software. Moreover, negative correlations were not considered in the simulations.

The autocorrelation of r_t in simulations 2 and 3 do not necessarily entail an autocorrelation of X. While this could in principle be added to the simulation model, it would mean there is a greater correlation between the time series in X that is not known by the parameter r_t. This caveat does not however impact the connectivity between the two signals.

Finally, it is not possible to tweak the auto-correlation of the time-series in simulations 2-3 without affecting the underlying correlation between the time series. This could be modified in future versions to make this parameter independent.

Supplementary References

1. Gordon EM, Laumann TO, Gilmore AW, Petersen SE, Nelson SM, Dosenbach NUF, et al. Precision Functional Mapping of Individual Human NeuroResource Precision Functional Mapping of Individual Human Brains. Neuron. Elsevier Inc. 2017; 1–17. doi:10.1016/j.neuron.2017.07.011

2. Shen X, Tokoglu F, Papademetris X, Constable R. Groupwise whole-brain parcellation from resting-state fMRI data for network node identification. NeuroImage. 2013;82: 403–415. doi:10.1016/j.neuroimage.2013.05.081

3. Thompson WH, Fransson P. On Stabilizing the Variance of Dynamic Functional Brain Connectivity Time Series. Brain Connectivity. 2016;6: 735–746. doi:10.1089/brain.2016.0454

4. Thompson WH, Fransson P. Bursty properties revealed in large-scale brain net-

works with a point-based method for dynamic functional connectivity. Scientific Reports. Nature Publishing Group; 2016;6: 39156. doi:10.1038/srep39156

5. Allen EA, Damaraju E, Plis SM, Erhardt EB, Eichele T, Calhoun VD. Tracking whole-brain connectivity dynamics in the resting state. Cerebral Cortex. 2014;24: 663–676. doi:10.1093/cercor/bhs352
