# Supplementary material for: Simulations to benchmark time-varying connectivity methods for fMRI
Source: PLoS Comput Biol. 2018 May 29;14(5):e1006196. doi: 10.1371/journal.pcbi.1006196 (PMC5993323; doi:10.1371/journal.pcbi.1006196)
Supplement: S3 Appendix — (PDF) [file pcbi.1006196.s003.pdf]

S3 Appendix: Varying the variance of r_t in simulation 2.

In simulation 2, the variance of the fluctuating covariance (σ_r) in the main text was set to 0.1. Simulation 2 was run with σ_r set to 0.08 and 0.12 as well. S3 Appendix, Figures AB show the posterior distributions of β for each TVC method (same as Fig. 6 in the main text). The evaluation of model fit is displayed in S3 Appendix, Table A-F.

Overall the results are similar to those in Fig. 6 in the main text. Here we can see that changing σ_r effects the magnitude of β but does not effect how the different methods perform. The reason for the magnitude of β increasing as σ_r increases is because r_t is varying more. This decreases the uncertainty of identifying r_t since the time series covariance is drawn from distributions where r_t is a parameter. If r_t varies more, it entails that draws from distributions with different r_t values are more dissimilar.

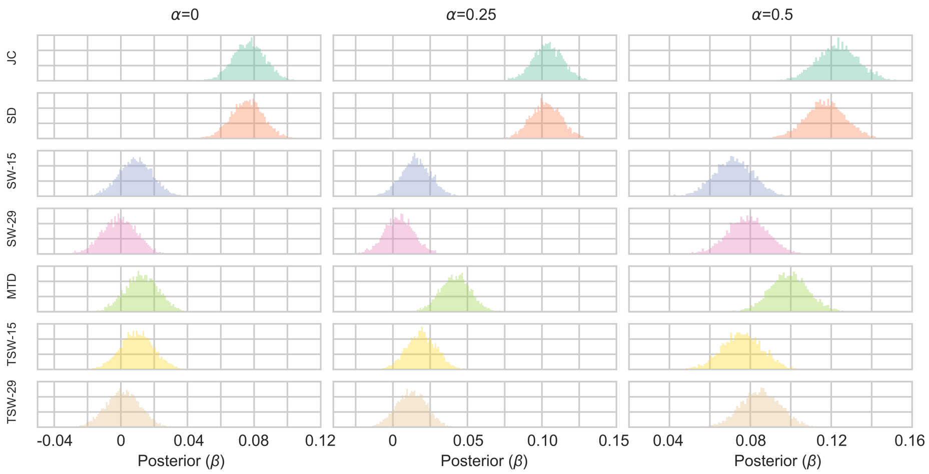

Figure A: Posterior distributions of the β parameter of the Bayesian linear regression models in Simulation 2. The figure shows the results for varying values of the autocorrelation parameter (α) where the variance of the fluctuating covariance (σ_r) is equal to 0.08. Compliments Fig. 6 in main text.

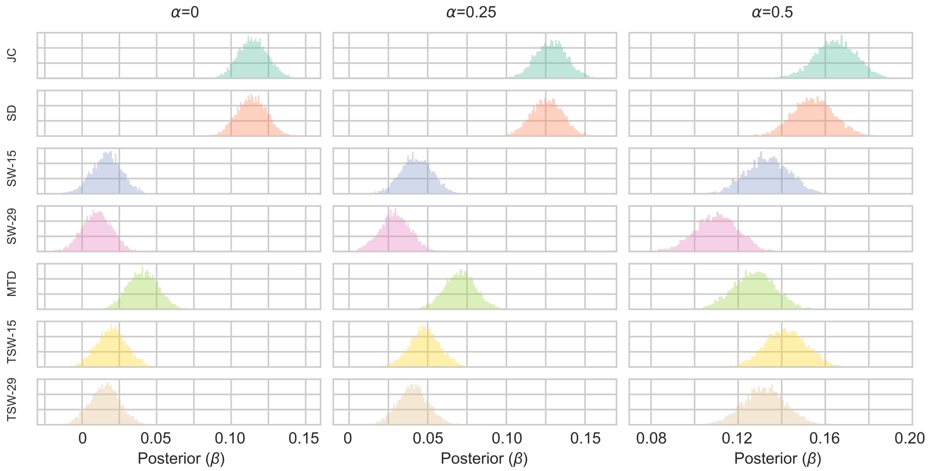

Figure B: Posterior distributions of the β parameter of the Bayesian linear regression models in Simulation 2. The figure shows the results for varying values of the autocorrelation parameter (α) where the variance of the fluctuating covariance (σ_r) is equal to 0.12. Compliments Fig. 6 in main text.

Table A: Results of Simulation 2 where $\alpha = 0.0$ and $\sigma_{r_t} = 0.08$. Tables shows WAIC, WAIC standard error, and difference in WAIC from the best performing method. A lower WAIC indicates a better fit.

Model	WAIC	WAIC SE	Δ WAIC
JC	28244.9	142.892	0
SD	28247	142.899	2.01576
MTD	28302.7	143.018	57.7415
TSW-15	28303.4	142.98	58.4582
SW-15	28303.5	142.974	58.5909
TSW-29	28304.4	143.02	59.4662
SW-29	28304.6	143.003	59.6281

Table B: Results of Simulation 2 where $\alpha = 0.0$ and $\sigma_{r_t} = 0.12$. Tables shows WAIC, WAIC standard error, and difference in WAIC from the best performing method. A lower WAIC indicates a better fit.

Model	WAIC	WAIC SE	Δ WAIC
JC	28172	141.092	0
SD	28174.2	141.028	2.25273
MTD	28287.7	141.207	115.766
TSW-15	28300.5	141.157	128.541
SW-15	28301.5	141.104	129.497
TSW-29	28302.1	141.132	130.127
SW-29	28303.5	141.212	131.521

Table C: Results of Simulation 2 where $\alpha = 0.25$ and $\sigma_{r_t} = 0.08$. Tables shows WAIC, WAIC standard error, and difference in WAIC from the best performing method. A lower WAIC indicates a better fit.

Model	WAIC	WAIC SE	Δ WAIC
JC	28195.6	141.754	0
SD	28198.5	141.853	2.92245
MTD	28286.7	141.931	91.1147
TSW-15	28300.7	141.838	105.12
SW-15	28301.9	141.795	106.334
TSW-29	28302.8	141.82	107.248
SW-29	28304.1	141.771	108.562

Table D: Results of Simulation 2 where $\alpha = 0.25$ and $\sigma_{r_t} = 0.12$. Tables shows WAIC, WAIC standard error, and difference in WAIC from the best performing method. A lower WAIC indicates a better fit.

Model	WAIC	WAIC SE	Δ WAIC
JC	28138.9	141.114	0
SD	28145.3	140.982	6.37803
MTD	28254.4	140.782	115.542
TSW-15	28281.1	140.996	142.241
SW-15	28285.9	141.052	147.029
TSW-29	28287.8	141.055	148.901
SW-29	28295.7	141.116	156.845

Table E: Results of Simulation 2 where $\alpha = 0.5$ and $\sigma_{r_t} = 0.08$. Tables shows WAIC, WAIC standard error, and difference in WAIC from the best performing method. A lower WAIC indicates a better fit.

Model	WAIC	WAIC SE	Δ WAIC
JC	28152	139.126	0
SD	28166.1	139.043	14.0644
MTD	28205.9	139.032	53.8909
TSW-29	28231.8	139.104	79.7723
SW-29	28242	138.986	90.0124
TSW-15	28246.3	139.075	94.3044
SW-15	28252.6	139.01	100.576

Table F: Results of Simulation 2 where $\alpha = 0.5$ and $\sigma_{r_t} = 0.12$. Tables shows WAIC, WAIC standard error, and difference in WAIC from the best performing method. A lower WAIC indicates a better fit.

Model	WAIC	WAIC SE	Δ WAIC
JC	28029.7	142.163	0
SD	28063.2	142.201	33.5364
TSW-15	28101	142.122	71.2716
SW-15	28124.7	142.106	94.9784
TSW-29	28128.8	142.219	99.1359
MTD	28140.9	142.147	111.182
SW-29	28183	142.237	153.295
